# HIF2α Promotes Cancer Metastasis through TCF7L2-Dependent Fatty Acid Synthesis in ccRCC

**DOI:** 10.34133/research.0322

**Published:** 2024-02-22

**Authors:** Jian Shi, Qingyang Lv, Daojia Miao, Zhiyong Xiong, Zhihao Wei, Songming Wu, Diaoyi Tan, Keshan Wang, Xiaoping Zhang

**Affiliations:** ^1^ Department of Urology, Union Hospital, Tongji Medical College, Huazhong University of Science and Technology, Wuhan 430022, Hubei, P. R. China.; ^2^ Institute of Urology, Union Hospital, Tongji Medical College, Huazhong University of Science and Technology, Wuhan 430022, Hubei, P. R. China.

## Abstract

Recent studies have highlighted the notable involvement of the crosstalk between hypoxia-inducible factor 2 alpha (HIF2α) and Wnt signaling components in tumorigenesis. However, the cellular function and precise regulatory mechanisms of HIF2α and Wnt signaling interactions in clear cell renal cell carcinoma (ccRCC) remain elusive. To analyze the correlation between HIF2α and Wnt signaling, we utilized the Cancer Genome Atlas - Kidney Renal Clear Cell Carcinoma (TCGA-KIRC) public database, HIF2α RNA sequencing data, and conducted luciferase reporter assays. A Wnt-related gene set was employed to identify key regulators of Wnt signaling controlled by HIF2α in ccRCC. Furthermore, we assessed the biological effects of TCF7L2 on ccRCC metastasis and lipid metabolism in both in vivo and in vitro settings. Our outcomes confirm TCF7L2 as a key gene involved in HIF2α-mediated regulation of the canonical Wnt pathway. Functional studies demonstrate that TCF7L2 promotes metastasis in ccRCC. Mechanistic investigations reveal that HIF2α stabilizes TCF7L2 mRNA in a method based on m^6^A by transcriptionally regulating METTL3. Up-regulation of TCF7L2 enhances cellular fatty acid oxidation, which promotes histone acetylation. This facilitates the transcription of genes connected to epithelial–mesenchymal transition and ultimately enhances metastasis of ccRCC. These outcomes offer a novel understanding into the involvement of lipid metabolism in the signaling pathway regulation, offering valuable implications for targeted treatment in ccRCC.

## Introduction

Renal cell carcinoma (RCC) is a prevalent urinary system malignancy [1], with clear cell RCC (ccRCC) as the predominant subtype [2]. Most ccRCC cases are connected with the inactivation of the cancer inhibitor gene von Hippel–Lindau (VHL) [3], which causes the dysregulation of hypoxia-inducible factors 1 alpha (HIF1α) and 2 alpha (HIF2α) through the ubiquitin-dependent degradation pathway [4]. However, while HIF1α inhibits the progression of ccRCC [5], HIF2α serves as an oncogene primarily by supporting the ccRCC occurrence and development through the transcriptional activation of downstream genes [6,7]. Extensive research has revealed that the aberrant HIF2α activation plays a key function in promoting cell survival and metastasis in ccRCC [8,9]. Therefore, targeting HIF2α is considered a pivotal therapeutic strategy for treating RCC [10,11].

Characterized by the accumulation of clear cytoplasm due to the presence of abundant intracellular lipids, ccRCC exhibits dysregulation of lipid metabolism that has a crucial function in its pathogenesis and development [12]. Altered lipid metabolism in ccRCC cells leads to increased lipid synthesis, aberrant lipid accumulation, and dysregulated lipid signaling, thereby enhancing cancer growth, invasion, and metastasis [13]. HIF2α has been discovered to have a function in promoting lipid accumulation in ccRCC cells by modulating the expression of crucial lipogenic enzymes and transporters, either directly or indirectly [14–16]. It enhances fatty acid uptake and stimulates de novo lipogenesis. Nevertheless, the specific mechanisms by which HIF2α-dependent increase in lipid synthesis and lipid storage contribute to ccRCC carcinogenesis remain unclear.

The initiation of Wnt signaling occurs via the connection between Wnt ligands and receptors situated on the cellular surface, resulting in the stimulation of the canonical (depending on β-catenin) or non-canonical (independent of β-catenin) mechanisms [17]. In the canonical pathway, Wnt signaling stabilizes β-catenin, enabling its translocation into the nucleus where it regulates the gene transcription related to cell growth, differentiation, and self-renewal of stem cell. The non-canonical pathway, on the other hand, influences processes such as cytoskeletal rearrangement, planar cell polarity, and calcium signaling [18,19]. Emerging evidence suggests that HIF2α has the capability to stimulate canonical Wnt signaling in tumors, establishing intricate crosstalk with the Wnt/β-catenin mechanism [20,21]. Transcription factor 7-like 2 (TCF7L2) is a member of the T-cell factor/lymphoid enhancer factor family and serves as a crucial transcription factor within the canonical Wnt signaling pathway. It constructs a compound with β-catenin and acts as a key regulator of downstream target genes, thereby facilitating tumor progression [22]. TCF7L2 is involved in cellular metabolic reprogramming, and studies have shown that TCF7L2 down-regulates hepatic gluconeogenesis while promoting lipid accumulation [23]. Additionally, TCF7L2 influences adipogenesis in adipose tissue [24]. Therefore, additional examination is warranted to unravel the molecular pathways through which TCF7L2 influences lipid metabolism in ccRCC cells.

Epithelial–mesenchymal transition (EMT) is a process characterized by morphological changes, where polarized epithelial cells undergo a transformation into spindle-shaped cells, acquiring a mesenchymal phenotype [25]. It is causally linked to cancer invasion, metastasis [26,27], and chemotherapy resistance [28]. In ccRCC, activation of EMT serves as the foundation for its malignant progression and metastatic dissemination [29–31]. Recent studies have highlighted the intertwined relationship between metabolic alterations and EMT. While metabolic changes can induce EMT, the process of EMT can also lead to metabolic alterations [32,33]. Abnormal lipid metabolism can activate EMT and promote tumor invasion and metastasis [34,35]. For instance, fatty acid translocase CD36 expression causes an increase in intracellular fatty acid concentrations and facilitates the process of EMT in hepatocellular carcinoma cells [36]. Genes involved in fatty acid uptake are frequently overexpressed in metastatic cancers and are connected with EMT in various malignancies [37]. Nevertheless, the relationship between lipid metabolism and EMT in ccRCC is still lacking in comprehensive understanding.

In summary, our research sheds light on the critical role of the HIF2α–METTL3–TCF7L2 axis in ccRCC progression. We demonstrate that HIF2α transcriptionally activates METTL3, leading to enhanced m^6^A modification of TCF7L2 mRNA, which promotes lipid synthesis and subsequent EMT activation. The exploration of these molecular mechanisms holds promise for the advancement of targeted therapies against ccRCC and potentially other malignancies that involve perturbed lipid metabolism and EMT, paving the way for innovative treatment strategies.

## Results

### TCF7L2 serves as a potential downstream gene regulated by HIF2α in the canonical Wnt pathway, exhibiting an elevated expression level in ccRCC

**Fig. 1.**
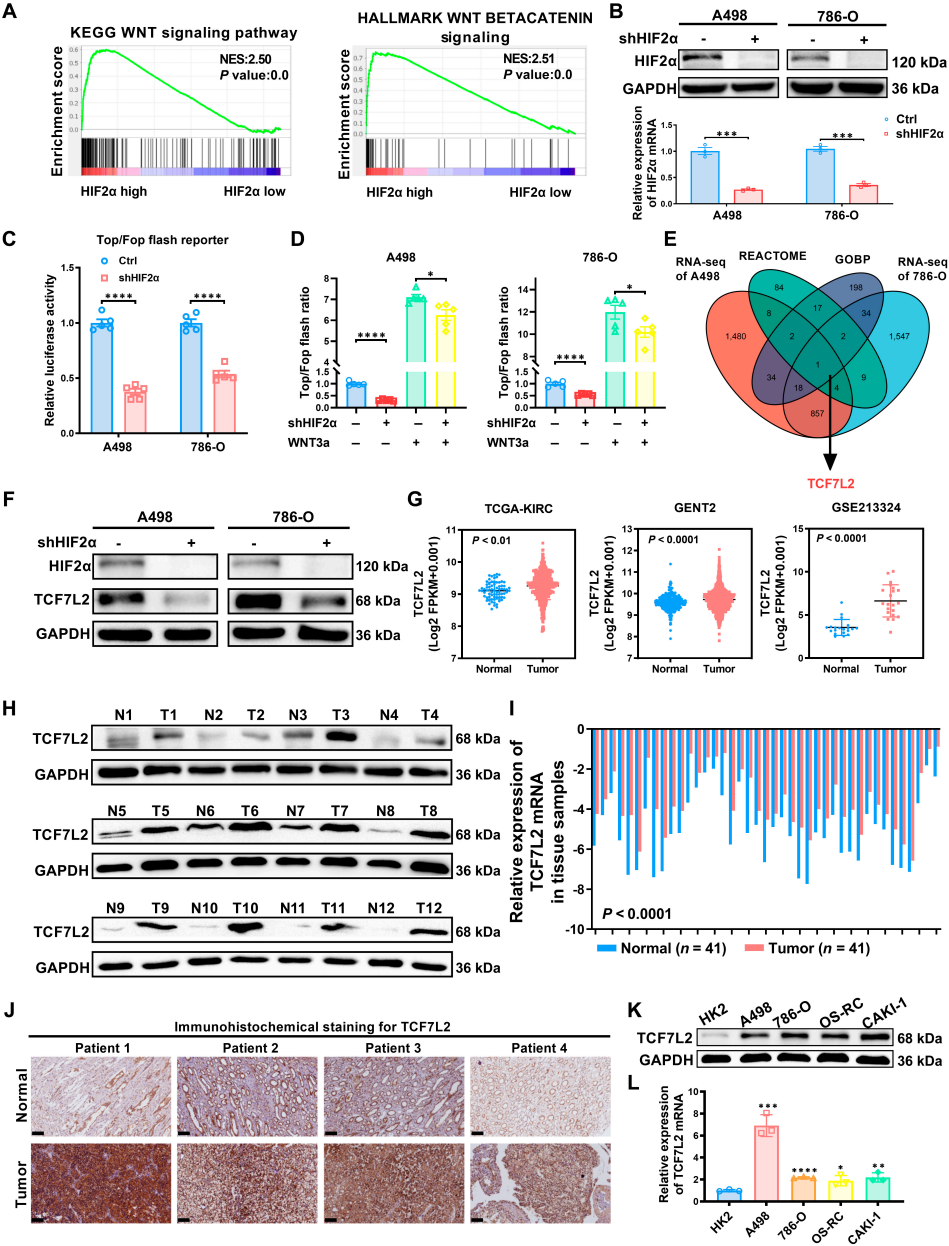
TCF7L2 serves as a potential downstream gene regulated by HIF2α in the canonical Wnt pathway, exhibiting an elevated expression level in ccRCC. (A) GSEA, depending on the TCGA database, reveals the association between HIF2α expression and the Wnt signaling mechanism, and the WNT BETA CATENIN signaling, in ccRCC. FDR < 25% and *P* < 0.05 were statistically significant. (B) The knockdown of HIF2α was validated at both the protein and mRNA levels utilizing Western blotting and qPCR, respectively. (C) In stable HIF2α knockdown ccRCC cells, luciferase reporter activity of TOP/FOP flash was measured after indicated treatment (*n* = 5). Statistical analysis was conducted utilizing independent-samples *t* test. (D) The luciferase reporter activity of TOP/FOP flash was detected in HIF2α knockdown ccRCC cells upon addition of Wnt3a (*n* = 5). Statistical analysis was conducted employing independent-samples *t* test. (E) A Venn diagram illustrating the overlap between 2 independent gene sets related to canonical Wnt signaling derived from the REACTOME database and the GOBP database, along with the whole transcriptome sequencing data acquired following stable HIF2α reduction. (F) Western blot analysis of HIF2α and TCF7L2 in ccRCC cells (*n* = 3). (G) The mRNA levels of TCF7L2 in healthy and ccRCC tissues were analyzed employing sequencing data from the TCGA-KIRC database, GENT2 database, and GEO database. *t* test, *P* < 0.0001 (independent-samples *t* test for overall variations analysis, paired-samples *t* test for N/T pairs analysis). (H) Levels of the TCF7L2 protein in ccRCC (*n* = 12) and adjacent normal tissues (*n* = 12). (I) TCF7L2 mRNA levels in 41 ccRCC and adjacent normal tissues (take the mRNA level of normal tissues as the control group). *t* test, *P* < 0.0001 (*n* = 41) (paired-samples *t* test for statistics). (J) Immunohistochemical staining of TCF7L2 in ccRCC and adjacent normal tissues (scale bar: 100 μm). (K) TCF7L2 protein levels in 4 ccRCC cell lines and one normal kidney cell line. (L) TCF7L2 mRNA levels in 4 ccRCC cell lines and one normal kidney cell line.

HIF2α is an oncogene associated with ccRCC [9]. It is strongly associated with the ccRCC cell transformation, leading to increased heterogeneity and invasive capabilities [38]. Targeting HIF2α and its downstream genes has been widely utilized in the treatment of metastatic ccRCC [39,40]. Through Gene Set Enrichment Analysis (GSEA) of HIF2α in the Cancer Genome Atlas (TCGA) database, we observed a notable enrichment of HIF2α in the canonical Wnt signaling mechanism (Fig. 1A). Consequently, we constructed HIF2α knockdown A498 and 786-O cell lines using shRNA (Fig. 1B) and performed whole transcriptome sequencing. We further elucidated the involvement and regulation of HIF2α in the Wnt pathway by conducting Gene Ontology (GO) analysis on the differentially expressed genes identified through whole transcriptome sequencing (Fig. S1A). To evaluate the Wnt pathway activation, we cloned 3 binding sites of TCF/LEF1 DNA (TOP-flash) or mutated TCF/LEF1 binding (FOP-flash) into luciferase reporter plasmids and transfected them into HIF2α knockdown ccRCC cells. Following HIF2α knockdown, the activity of TOP/FOP-flash was markedly inhibited (Fig. 1C). However, this inhibition was rescued upon treatment with the canonical Wnt ligand Wnt3a (Fig. 1D). These findings establish the stimulation of the established Wnt pathway by HIF2α. To ascertain the target genes regulated by HIF2α in the canonical Wnt mechanism, we performed screening of sequencing data from HIF2α knockdown in A498 and 786-O cells using 2 independent gene sets associated with canonical Wnt signaling. Notably, TCF7L2 showed prominent differential expression in ccRCC (Fig. 1E). Depending on RNA-seq data from the Cancer Genome Atlas - Kidney Renal Clear Cell Carcinoma (TCGA-KIRC) database, we generated a linear correlation curve between TCF7L2 and HIF2α. The outcomes exhibited a significant positive relationship between TCF7L2 and HIF2α (Fig. S1B). Western blot and qPCR results demonstrate that, following the knockdown of HIF2α, the expression of TCF7L2 decreases (Fig. 1F and Fig. S1C). Conversely, under hypoxic conditions, the up-regulation of HIF2α markedly increases the expression of TCF7L2 (Fig. S1D). This indicates that TCF7L2 is a downstream gene of HIF2α. We confirmed the up-regulation of TCF7L2 at both the mRNA and protein levels in ccRCC through several databases (Fig. 1G and Fig. S1E). Furthermore, in terms of clinical and pathological parameters of ccRCC, the expression of TCF7L2 showed a high predictive value (Fig. S1F). Receiver operating characteristic (ROC) curve analysis indicates that the expression of TCF7L2 has notable diagnostic value for the occurrence and metastasis of ccRCC (Fig. S1G and H). TCF7L2 expression analysis in ccRCC patient samples demonstrated elevated levels of TCF7L2 protein and mRNA in ccRCC tissues in contrast to adjacent tissues (Fig. 1H and J and Fig. S1I). Moreover, similar results were detected in cell lines of ccRCC (Fig. 1K and L).

### TCF7L2 promotes metastasis and EMT in ccRCC

**Fig. 2.**
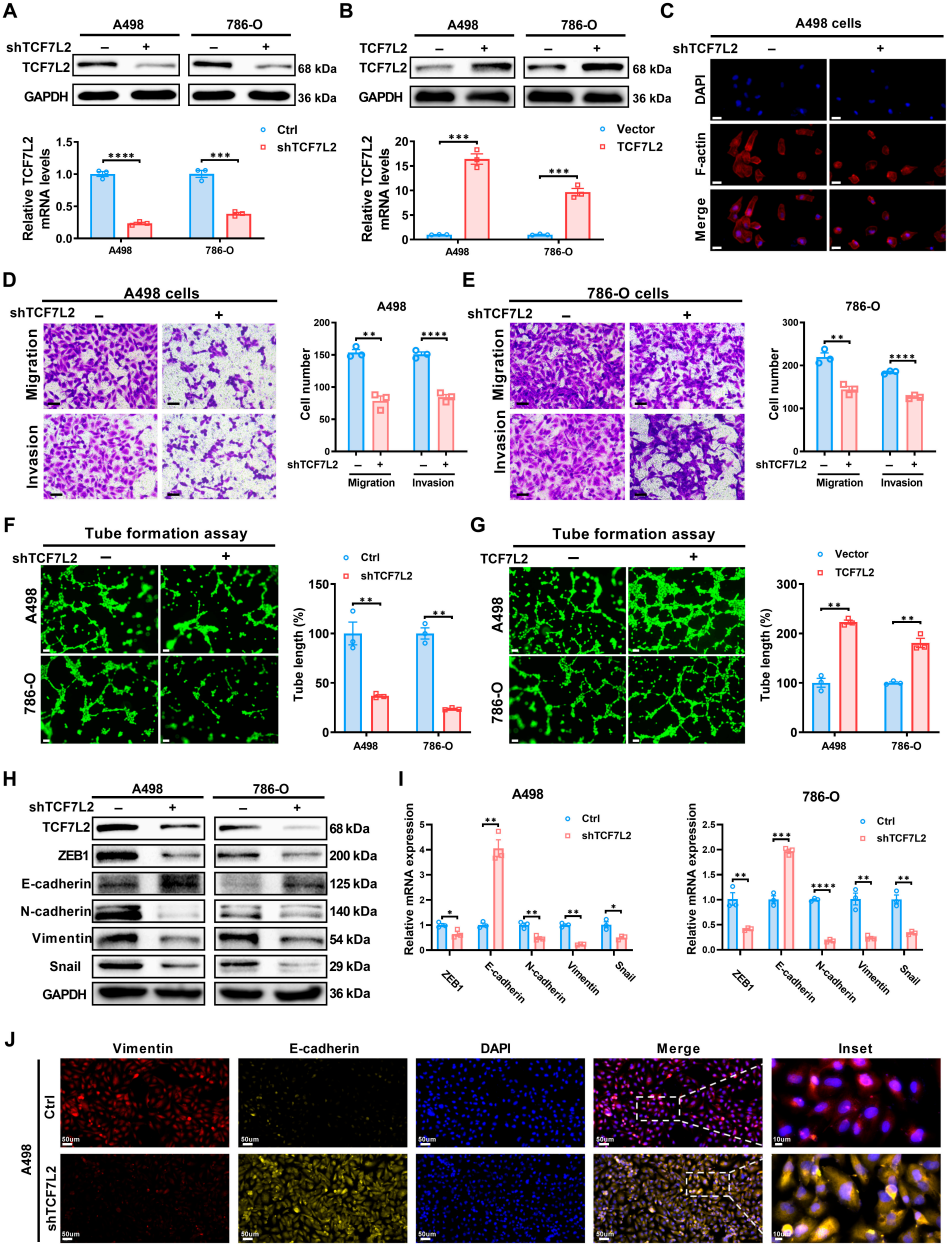
TCF7L2 promotes metastasis and EMT in ccRCC. Knockdown of TCF7L2 or ccRCC cell line overexpression was established by infecting with shRNA and overexpression lentiviruses, respectively. The results represent the mean ± SEM of 3 independent trials with at least 3 replications per trial. ****P < 0.0001, ***P < 0.001, **P < 0.01, and *P < 0.05. (A) The knockdown of TCF7L2 was validated at both the protein and mRNA levels using Western blotting and qPCR, respectively. (B) The TCF7L2 overexpression was validated at both the protein and mRNA levels employing Western blotting and qPCR, respectively. (C) Cell nuclei and cytoskeleton were stained using DAPI and F-actin, respectively (*n* = 3, scale bar: 50 μm). (D) Transwell assay results indicating the migration and invasion of TCF7L2 knockdown A498 cells (*n* = 3, scale bar: 50 μm). Statistical analysis was conducted employing independent-samples *t* test. (E) Transwell assay results showing the migration and invasion of TCF7L2 knockdown 786-O cells (*n* = 3, scale bar: 50 μm). Statistical analysis was conducted employing independent-samples *t* test. (F) Effect of TCF7L2 knockdown in ccRCC cells on tube formation assay in HUVECs (*n* = 3). Statistical analysis was performed employing independent-samples *t* test. (G) Effect of TCF7L2 overexpression in ccRCC cells on tube formation assay in HUVECs (*n* = 3). Statistical analysis was performed employing independent-samples *t* test. (H) Western blot analysis of EMT biomarkers in TCF7L2 knockdown ccRCC cells (*n* = 3). (I) qPCR analysis of EMT biomarkers in TCF7L2 knockdown ccRCC cells (*n* = 3). Statistical analysis was conducted employing independent-samples *t* test. (J) Immunofluorescence staining of vimentin, E-cadherin, and DAPI in TCF7L2 knockdown ccRCC cells (*n* = 3, scale bar: 50 μm).

The HIF2α function in regulating cell migration, growth, and invasion has been demonstrated in ccRCC [41]. The marked up-regulation of TCF7L2, as a downstream gene of HIF2α, in ccRCC suggests its probable function in disease development. Due to the activation of HIF2α in ccRCC being caused by VHL mutations, we selected the ccRCC cell lines A498 and 786-O with VHL mutations for subsequent studies. To elucidate the impact of TCF7L2 on the ccRCC biological functions, stable cell lines with TCF7L2 knockdown and overexpression were established in A498 and 786-O cells using lentiviral infection with TCF7L2 shRNA or overexpression vectors (Fig. 2A and B). Results from CCK-8 cell viability analysis revealed that alterations in TCF7L2 expression levels did not affect the proliferative capacity of ccRCC cells (Fig. S2A and B). To examine cellular cytoskeletal structure, F-actin was stained with phalloidin. TCF7L2 knockdown cells exhibited a more rounded morphology and reduced stress fibers compared to control cells (Fig. 2C), whereas TCF7L2 overexpression cells displayed enhanced front–rear polarization and F-actin rearrangement (Fig. S2C), indicating increased cellular invasive ability. Transwell assays validated that TCF7L2 knockdown markedly attenuated cell migration and invasion (Fig. 2D and E). Conversely, TCF7L2 overexpression enhanced the abilities of A498 and 786-O cells to migrate and invade (Fig. S2D). Consistent results were obtained in the scratch wound healing assay (Fig. S2E–H). Furthermore, tube formation assays revealed that TCF7L2 knockdown inhibited the human umbilical vein endothelial cell (HUVEC) network construction (Fig. 2F), whereas TCF7L2 overexpression showed the opposite effect (Fig. 2G). To elucidate whether TCF7L2 also exerts its effects in VHL wild-type ccRCC cells, stable cell lines with TCF7L2 knockdown and overexpression were generated in CAKI cells (Fig. S3A and B). Transwell results indicated that TCF7L2 knockdown partially inhibited the migration and invasion of CAKI cells, while TCF7L2 overexpression promoted the migration and invasion of CAKI cells (Fig. S3C and D). Tube formation assay results revealed that TCF7L2 knockdown partially suppressed network formation in HUVECs, whereas TCF7L2 overexpression had the opposite effect (Fig. S3E and F). Although the functional impact of TCF7L2 on both VHL-mutant and wild-type ccRCC cell lines was similar, its regulatory role in VHL wild-type cell lines was relatively mild compared to its effects in VHL-mutant ccRCC cell lines. This suggests that the effects of TCF7L2 in ccRCC is primarily regulated by HIF2α, with the possibility of some additional alternative regulatory pathways.

These alterations in cellular morphology and invasiveness indicate that TCF7L2 expression induces EMT in the cells. Compared to control cells, TCF7L2 knockdown cells exhibited an increase in both protein and mRNA levels of E-cadherin, while ZEB1, N-cadherin, Vimentin, and Snail were decreased (Fig. 2H and I). Immunofluorescence analysis revealed reduced vimentin expression and increased E-cadherin-mediated adhesive junctions in TCF7L2 knockdown cells (Fig. 2J). These features indicate that TCF7L2 knockdown inhibits EMT in ccRCC cells. Conversely, overexpression of TCF7L2 down-regulated E-cadherin expression and up-regulated the ZEB1, Vimentin, N-cadherin, and Snail expression (Fig. S3G to I). In summary, TCF7L2 enhances cellular invasion, migration, and EMT occurrence in ccRCC.

### TCF7L2 promotes fatty acid synthesis and oxidation in ccRCC

**Fig. 3.**
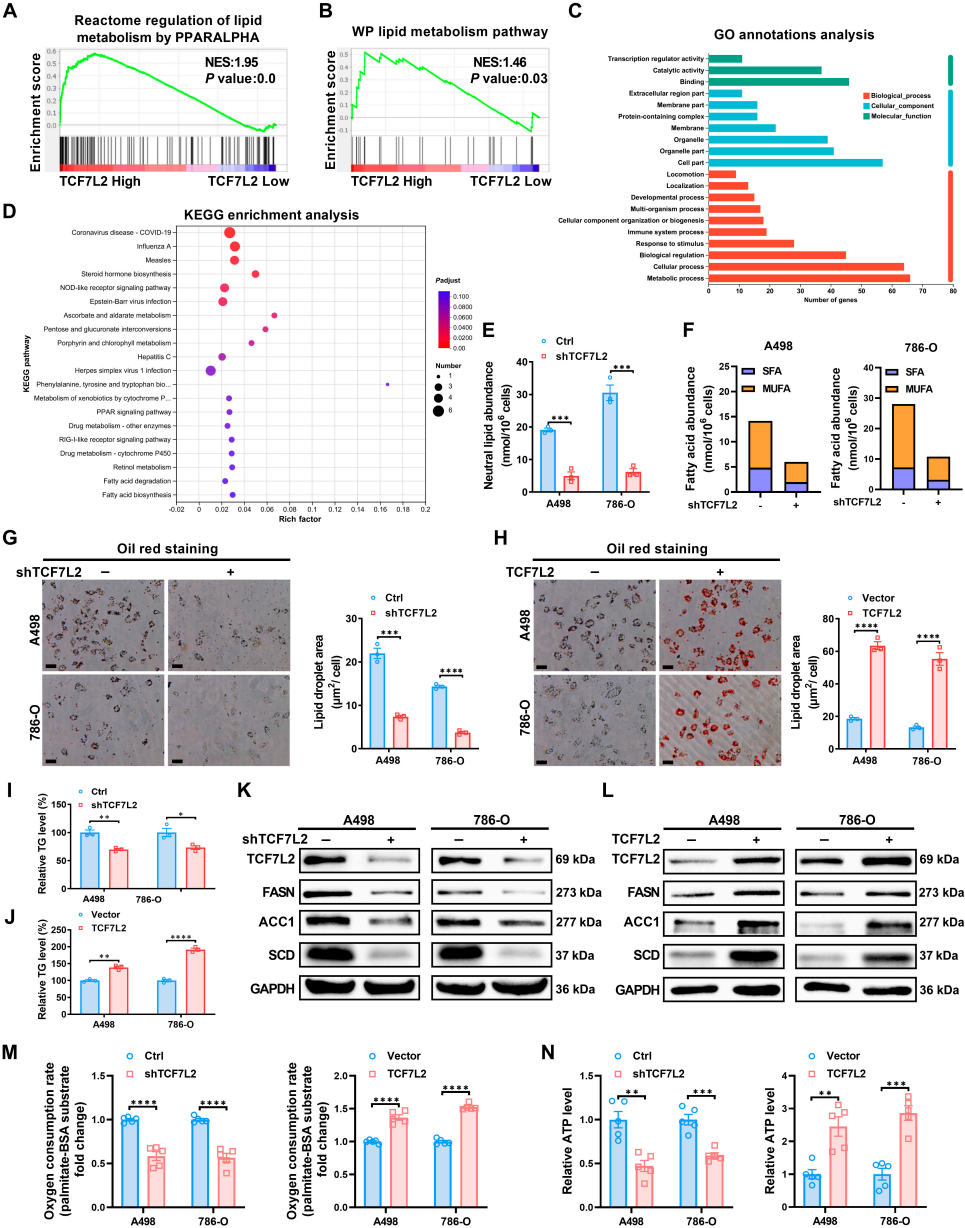
TCF7L2 promotes fatty acid synthesis and oxidation in ccRCC. The results represent the mean ± SEM of 3 independent trials with at least 3 replications per trial. ****P < 0.0001, ***P < 0.001, **P < 0.01, and *P < 0.05. (A and B) GSEA dependent on the TCGA database reveals the association between TCF7L2 expression and the regulation of lipid metabolism by PPARALPHA, as well as the lipid metabolism pathway, in ccRCC. FDR < 25% and *P* < 0.05 were statistically significant. (C) GO annotations analysis of differentially expressed genes determined by transcriptome sequencing. (D) KEGG enrichment analysis of differentially expressed genes determined by transcriptome sequencing. (E) Abundance of neutral lipids in TCF7L2 knockdown ccRCC cells (*n* = 3). Statistical analysis was conducted employing independent-samples *t* test. (F) Abundance of saturated fatty acids (SFA) and monounsaturated fatty acids (MUFA) in the neutral lipid fraction of TCF7L2 knockdown ccRCC cells. (G) Images and quantification of Oil Red O staining in TCF7L2 knockdown ccRCC cell lines compared to the negative control (*n* = 3, scale bar: 20 μm). (H) Images and quantification of Oil Red O staining in TCF7L2 overexpression ccRCC cell lines in contrast to the negative control (*n* = 3, scale bar: 20 μm). Statistical analysis was conducted employing independent-samples *t* test. (I and J) Relative levels of TG in TCF7L2 knockdown and overexpressing ccRCC cell lines. Statistical analysis was conducted employing independent-samples *t* test. (K and L) Western blot analysis of lipid synthesis gene proteins (FASN, ACC1, and SCD) in ccRCC cell lines with TCF7L2 knockdown or overexpression (*n* = 3). Statistical analysis was conducted employing independent-samples *t* test. (M) Measurement of OCR levels in the specified group of ccRCC cells and (N) intracellular ATP levels (*n* = 3). Statistical analysis was conducted employing independent-samples *t* test.

Given the impact of TCF7L2 on ccRCC metastasis, we aimed to elucidate its specific functional role in ccRCC (Fig. 3A and B). Therefore, we conducted GSEA on TCF7L2 in the database of TCGA, revealing its involvement in lipid metabolism modulation in ccRCC. Importantly, dysregulated lipid metabolism, as a prominent feature [42], serves as a crucial factor in the ccRCC development mediated by HIF2α [6]. Furthermore, we performed whole transcriptome sequencing on TCF7L2-knockdown A498 cells and performed GO and KEGG analyses on the differentially expressed genes. The outcomes indicated the involvement of TCF7L2 in cellular metabolic processes and its association with fatty acid synthesis and degradation (Fig. 3C and D and Fig. S3A and B). Lipidomics analysis verified a reduction in neutral lipids in ccRCC cells upon TCF7L2 knockdown (Fig. 3E), primarily characterized by a decrease in saturated and monounsaturated fatty acids (FAs) (Fig. 3F), while cholesterol esters showed no marked changes (Fig. S3C). Oil Red O staining showed decreased lipid content in TCF7L2 knockdown ccRCC cell lines **(**Fig. 3G**)**, while TCF7L2 overexpression resulted in increased lipid content (Fig. 3H). To assess the triglyceride (TG) content in TCF7L2-knockdown and overexpressing ccRCC cell lines, we measured cellular TG levels. The results showed that TCF7L2 knockdown decreased cellular TG content (Fig. 3I), while TCF7L2 overexpression increased TG levels (Fig. 3J), consistent with the Oil Red O staining results. Additionally, TCF7L2 knockdown or overexpression had no impact on cellular total cholesterol (TCH) levels (Fig. S3D and E). The reduction of TCF7L2 led to a notable down-regulation of FASN, ACC1, and SCD protein levels, which are vital genes related to fatty acid biosynthesis, as confirmed by qPCR and Western blot analysis (Fig. S3F and Fig. 3K), while TCF7L2 overexpression showed the opposite effect (Fig. S3G and Fig. 3L). These results confirm the promotive role of TCF7L2 in fatty acid synthesis in ccRCC. Subsequently, we evaluated long-chain fatty acid oxidation (FAO) using a palmitate ester oxidation stress test by measuring changes in extracellular oxygen consumption rate (OCR). Interestingly, TCF7L2 knockdown was associated with decreased OCR and intracellular adenosine triphosphate (ATP) levels, while TCF7L2 overexpression showed the opposite effect (Fig. 3M and N), suggesting a potential link to increased fatty acid synthesis. These findings support the role of TCF7L2 in up-regulating fatty acid uptake and inducing activation of the FAO pathway.

### EMT is controlled by TCF7L2-driven fatty acid metabolism

**Fig. 4.**
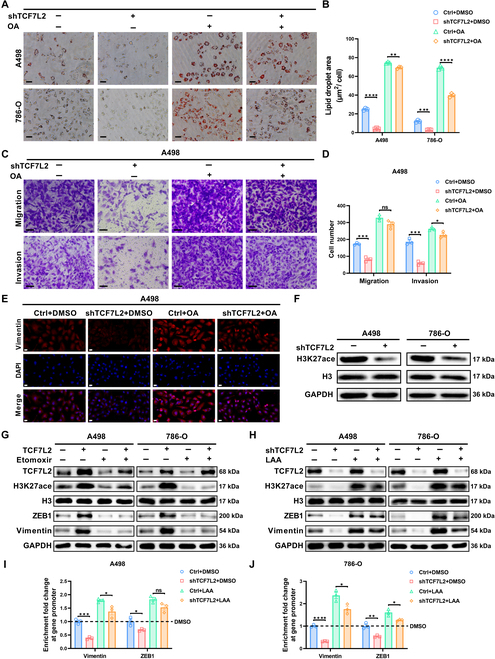
EMT is controlled by TCF7L2-driven fatty acid metabolism. The results represent the mean ± SEM of 3 independent trials with at least 3 replicates per trial. ****P < 0.0001, ***P < 0.001, **P < 0.01, and *P < 0.05. (A and B) Oil Red O staining images and measurement of Ctrl+DMSO, shTCF7L2+DMSO, Ctrl+OA, shTCF7L2+OA ccRCC cells (*n* = 3, scale bar: 20 μm). (C and D) Transwell assay results showing the Ctrl+DMSO, shTCF7L2+DMSO, Ctrl+OA, and shTCF7L2+OA A498 cell migration and invasion (*n* = 3, scale bar: 50 μm). Statistical analysis was conducted employing independent-samples *t* test. (E) Immunofluorescence staining of vimentin and DAPI of Ctrl+DMSO, shTCF7L2+DMSO, Ctrl+OA, and shTCF7L2+OA A498 cells (*n* = 3, scale bar: 20 μm). (F) Protein expression of H3K27 acetylation in TCF7L2 knockdown and negative control ccRCC cells. (G) Protein expression of H3K27 acetylation, ZEB1, and Vimentin in TCF7L2 overexpression and negative control ccRCC cells treated with DMSO and Etomoxir. (H) Protein expression of H3K27 acetylation, ZEB1, and Vimentin in TCF7L2 knockdown and negative control ccRCC cells treated with DMSO and LAA. (I and J) qPCR analysis of the EMT gene promoter region in ChIP samples of H3K27 acetylation in A498 cells or 786-O treated with LAA. Statistical analysis was conducted employing independent-samples *t* test.

We further investigated whether the increased fatty acid synthesis driven by TCF7L2 could serve as a prerequisite for the invasion of ccRCC cells. To establish a model of lipid overload, we treated TCF7L2 knockdown and negative control ccRCC cells with oleic acid (OA), a strong inducer of TG formation [43]. Following the addition of OA, the decrease in lipid content caused by TCF7L2 knockdown in ccRCC cells was rescued (Fig. 4A and B), indicating that TCF7L2 primarily influences ccRCC lipid metabolism by promoting TG synthesis. TCF7L2 knockdown-mediated suppression of ccRCC cell migration and invasion was reversed upon OA-induced increase in TG content, as demonstrated by Transwell assays (Fig. 4C and D and Fig. S4A). Additionally, immunofluorescence and qPCR results suggested that lipid accumulation could rescue the suppressive impact of TCF7L2 knockdown on EMT (Fig. 4E and Fig. S4B and C). These findings confirm that TCF7L2-activated EMT is driven by increased fatty acid synthesis. Additionally, we measured cellular OCR levels and found that increased fatty acid availability could rescue the decreased FAO levels caused by TCF7L2 knockdown in ccRCC cells (Fig. S4D), supporting the notion that TCF7L2’s activation of FAO in ccRCC is also influenced by lipid accumulation.

The main product of FAO is acetyl-CoA [44]. Acetyl-CoA, as a biosynthetic intermediate, is a determining factor in histone acetylation [45]. To elucidate the regulation of EMT gene transcription by fatty acid metabolism, we hypothesized that it may be controlled through epigenetic events of histone acetylation. Therefore, we analyzed the levels of histone acetylation after TCF7L2 knockdown. The acetylation levels of H3K14, H3K18, and H3K27, which control active gene transcription, were moderately reduced, with the most notable decrease observed in H3K27 acetylation (Fig. 4F and Fig. S4E). To further demonstrate that the levels of histone acetylation and EMT signaling are regulated by TCF7L2-mediated FAO, we inhibited cellular FAO levels in ccRCC cells overexpressing TCF7L2 by adding etomoxir. Western blot results indicated that after suppressing FAO levels, the up-regulation of TCF7L2-induced histone acetylation and activation of EMT were markedly restored (Fig. 4G). This suggests that TCF7L2 primarily promotes histone H3K27 acetylation and EMT signaling through the regulation of FAO. To further investigate the role of acetyl-CoA in this process, we supplemented lithium acetoacetate (LAA), an exogenous acetyl-CoA precursor, in TCF7L2-knockdown ccRCC cells. Western blot results demonstrated that the addition of acetyl-CoA rescued the inhibitory effect of TCF7L2 knockdown on histone H3K27 acetylation and EMT signaling (Fig. 4H). This confirms that the reduction of acetyl-CoA induced by TCF7L2 knockdown is a critical factor in the suppression of H3K27 acetylation and EMT signaling. To define whether the H3K27ac levels decrease leads to chromatin modifications at EMT gene promoters, chromatin immunoprecipitation (ChIP) was performed for H3K27ac in the existence or lack of LAA supplementation. In TCF7L2 knockdown ccRCC cells, a reduced enrichment of H3K27ac was observed at EMT gene promoters, which was rescued by LAA supplementation (Fig. 4I and J). Overall, the up-regulation of cellular fatty acid levels by TCF7L2 promotes an increase in FAO and contributes to the maintenance of H3K27 acetylation on EMT gene promoters, thereby facilitating the EMT process.

### TCF7L2 mediates the effects of HIF2α on ccRCC metastasis and lipid metabolism

**Fig. 5.**
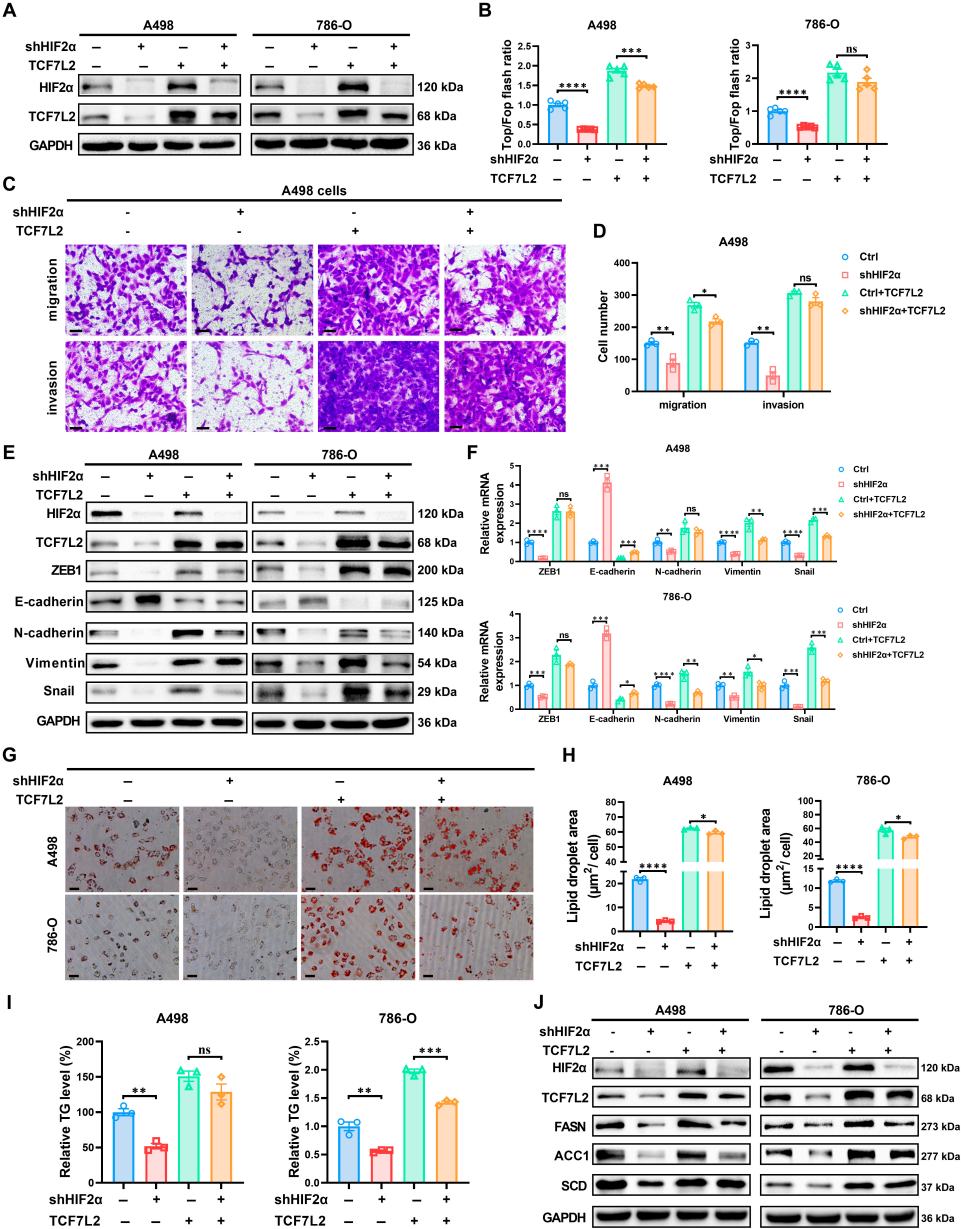
TCF7L2 mediates the HIF2α impacts on ccRCC metastasis and lipid metabolism. Four stable cell lines of ccRCC were created by infecting HIF2α-stably knocked down ccRCC cells with lentivirus overexpressing TCF7L2, resulting in the following groups: Ctrl (control), shHIF2α (HIF2α knocked down), Ctrl+TCF7L2 (control with TCF7L2 overexpression), and shHIF2α+TCF7L2 (HIF2α knocked down with TCF7L2 overexpression). Results represent the mean ± SEM of 3 independent trials with a minimum of 3 replications per trial. ****P < 0.0001, ***P < 0.001, **P < 0.01, and *P < 0.05. (A) Western blot analysis revealed the HIF2α and TCF7L2 protein levels in the stated cell lines (*n* = 3). (B) The luciferase reporter gene activity of TOP/FOP flash in the mentioned cell lines was assessed (*n* = 3). (C) Transwell assay images of the mentioned A498 cell lines (*n* = 3, scale bar: 50 μm). (D) Quantification of Transwell assay for the mentioned A498 cell lines (*n* = 3). Statistical analysis was conducted employing independent-samples *t* test. (E) Western blot analysis of EMT markers in the mentioned cell lines (*n* = 3). (F) qPCR analysis of EMT biomarkers in the mentioned cell lines (*n* = 3). Statistical analysis was conducted employing independent-samples *t* test. (G and H) Oil Red O staining images and quantification of the mentioned cell lines (*n* = 3). Statistical analysis was conducted employing independent-samples *t* test. (I) Relative levels of TG in the mentioned cell lines (*n* = 3). Statistical analysis was conducted employing independent-samples *t* test. (J) Western blot analysis of lipid synthesis-related genes FASN, ACC1, and SCD in the mentioned cell lines (*n* = 3).

We have confirmed the activating role of HIF2α on the canonical Wnt signaling. Considering that TCF7L2 is downstream of HIF2α depending on HIF2α transcriptome sequencing results and the canonical Wnt signaling pathway screening, we hypothesize that TCF7L2 mediates the stimulation of the canonical Wnt signaling and the metastatic effects of HIF2α in ccRCC. To validate this hypothesis, we constructed a functional rescue model in HIF2α-knockdown ccRCC cells using TCF7L2 overexpression lentivirus (Fig. 5A and Fig. S5A). In order to clarify whether HIF2α activates the canonical Wnt signaling pathway through TCF7L2, we performed a luciferase reporter gene assay using TOP/FOP-flash in ccRCC cells from the functional rescue model. After HIF2α knockdown, the activity of TOP/FOP-flash was markedly inhibited, and this inhibition was rescued by TCF7L2 overexpression (Fig. 5B). Prior investigations have revealed the carcinogenic role of HIF2α in promoting ccRCC development and metastasis [46,47]. In this investigation, we also detected a notable reduction in ccRCC cells’ capability to migrate and invade upon HIF2α knockdown. Nevertheless, TCF7L2 overexpression alleviated this inhibition (Fig. 5C and D and Fig. S5B and C). Subsequently, we investigated the activation of EMT signaling. HIF2α knockdown caused the down-regulation of ZEB1, Vimentin, N-cadherin, and Snail, along with E-cadherin up-regulation. The overexpression of TCF7L2 reversed this effect (Fig. 5E and F). Next, we explored the changes in lipid metabolism in the corresponding ccRCC cells. Clearly, TCF7L2 overexpression rescued the suppressive impact of HIF2α reduction on lipid (Fig. 5G and H), TG (Fig. 5I), and FAO (Fig. S5D) levels in ccRCC cells. After HIF2α knockdown, the protein and mRNA levels of lipid synthesis-related genes FASN, ACC1, and SCD were down-regulated, and this down-regulation was rescued by TCF7L2 overexpression (Fig. 5J and Fig. S5E and F). These findings suggest that TCF7L2 serves as a major downstream gene through which HIF2α promotes ccRCC metastasis and regulates lipid metabolism.

### HIF2α transcriptionally activates METTL3 to stabilize TCF7L2 mRNA

**Fig. 6.**
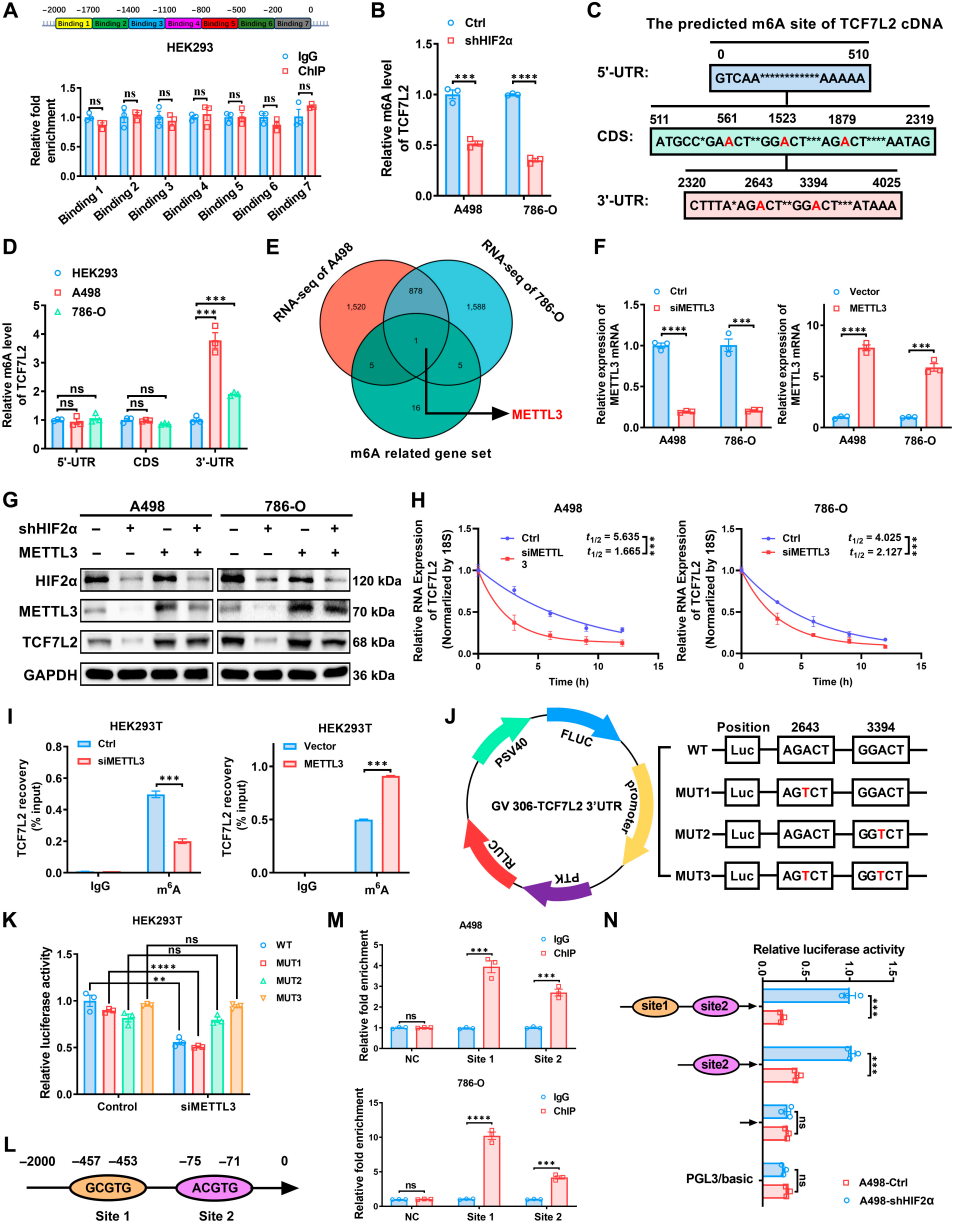
HIF2α transcriptionally activates METTL3 to stabilize TCF7L2 mRNA. The results represent the mean ± SEM of 3 independent trials with minimum 3 replications per trial. ****P < 0.0001, ***P < 0.001, **P < 0.01, and *P < 0.05. (A) qPCR analysis of Binding 1 to Binding 7 based on ChIP experiments (*n* = 3). Statistical analysis was conducted employing independent-samples *t* test. (B) Levels of m^6^A modification in TCF7L2 in HIF2α knockdown and control ccRCC cells (*n* = 3). Statistical analysis was conducted employing independent-samples *t* test. (C) Diagram of m^6^A modification sites in TCF7L2 predicted using SRAMP. The red "A" represents potential modification sites. (D) m^6^A modification levels in various sites of TCF7L2 between ccRCC and normal cell lines (*n* = 3). Statistical analysis was conducted employing independent-samples *t* test. (E) Venn diagram demonstrating the overlapping genes between differentially expressed genes in the HIF2α knockdown transcriptome and m^6^A-related genes. METTL3 is the only gene found in the intersection. (F) qPCR analysis of METTL3 in METTL3 knockdown or overexpression ccRCC cell lines (*n* = 3). Statistical analysis was conducted employing independent-samples *t* test. (G) Western blot analysis of METTL3 and TCF7L2 in HIF2α knockdown and METTL3 overexpression ccRCC cell lines (*n* = 3). (H) Decay rates of TCF7L2 mRNA after METTL3 knockdown in A498 and 786-O cells treated with Actinomycin D (*n* = 3). Statistical analysis was conducted employing independent-samples *t* test. (I) MeRIP analysis of TCF7L2 mRNA using m^6^A antibody in METTL3- knockdown or overexpressed HEK293T cells (*n* = 3). Statistical analysis was conducted employing independent-samples *t* test. (J) Schematic diagram depicting the specific site mutations and luciferase reporter gene vectors of wild-type TCF7L2 3′-UTR and mutant 3′-UTR. (K) Relative luciferase activity of wild-type and mutant TCF7L2 3′-UTR reporter vectors following METTL3 knockdown (*n* = 3). Statistical analysis was conducted employing independent-samples *t* test. (L) Schematic representation of probable HIF2α binding regions in the TCF7L2 promoter. (M) qPCR analysis of site 1 and site 2 based on ChIP experiments (*n* = 3). Statistical analysis was conducted employing independent-samples *t* test. (N) Relative luciferase activity of the truncated plasmids constructed based on site 1 and site 2 in A498 cells (*n* = 3). Statistical analysis was conducted employing independent-samples *t* test.

HIF2α, as a transcription factor, primarily exerts its regulatory role by transcriptionally activating downstream target genes [48,49]. Transcriptomic sequencing data indicate TCF7L2 as a downstream gene of HIF2α, and a positive correlation between TCF7L2 and HIF2α is observed (Fig. 1F and Fig. S1B and C). Initially, we considered the possibility of HIF2α directly transcribing and activating TCF7L2 mRNA expression. However, analysis of the promoter region upstream of the TCF7L2 transcription start site within 2,000 bp did not reveal hypoxia response elements (HREs) for HIF2α, suggesting that HIF2α may not directly bind to the TCF7L2 promoter. To further validate this hypothesis, we divided the TCF7L2 promoter into 7 binding regions, and ChIP results indicated that HIF2α cannot bind to the TCF7L2 promoter, thus negating the possibility of direct transcriptional activation (Fig. 6A and Fig. S7A). Consequently, we investigated the influence of HIF2α reduction on the pre-mRNA levels of TCF7L2. Interestingly, our findings suggest that HIF2α does not modulate TCF7L2 expression by affecting its pre-mRNA levels (Fig. S8A). This suggests that TCF7L2 is regulated by HIF2α post-transcriptionally. RNA modification and degradation are crucial processes in post-transcriptional regulation, and considering m^6^A as the most prevalent RNA modification in humans [50], we initially investigated HIF2α’s impact on m^6^A modification of TCF7L2 mRNA through methylated RNA immunoprecipitation (MeRIP)-qPCR analysis. The results confirmed a reduction in m^6^A modification levels in TCF7L2 mRNA in ccRCC cells with HIF2α knockdown (Fig. 6B). Sequence-based m^6^A modification site predictor (SRAMP) prediction based on the TCF7L2 cDNA sequence identified 5 potential m^6^A sites (Fig. 6C and Table S4). Additionally, we discovered that the increased m^6^A modification levels in tumor cell TCF7L2 were primarily enriched in the 3′-UTR (Fig. 6D), which aligns with the prevailing notion that most m^6^A residues exhibit 3′-UTR localization bias and are associated with mRNA instability [51]. We performed a screening of m^6^A-regulated genes in the transcriptomic sequencing results of HIF2α knockdown cells, which led us to identify METTL3 (Fig. 6E). METTL3 is an m^6^A RNA methyltransferase known to regulate mRNA stability [52]. The down-regulation of METTL3 expression in HIF2α knockdown cells was subsequently confirmed through Western blotting and qPCR analysis (Fig. S8B and C). We established METTL3 knockdown and overexpression ccRCC cell lines using siRNA and overexpression plasmids, respectively (Fig. 6F and Fig. S8D). Western blotting and qPCR analysis indicated that reduced METTL3 levels caused up-regulated TCF7L2 expression, whereas overexpression of METTL3 increased TCF7L2 expression (Fig. S8E and F).We further established stable cell lines overexpressing METTL3 in ccRCC cells with stable HIF2α knockdown. Results from Western blotting and qPCR analyses indicated that the up-regulation of METTL3 rescued the inhibitory effect of HIF2α knockdown on TCF7L2 expression (Fig. 6G and Fig. S8G). This suggests that the regulation of TCF7L2 by HIF2α is mediated through METTL3. To clarify whether the regulatory effect of METTL3 on ccRCC metastasis primarily depends on TCF7L2, we overexpressed TCF7L2 in stable METTL3-knockdown ccRCC cell lines (Fig. S1A and B). Results from Transwell experiments showed that the overexpression of TCF7L2 reversed the inhibitory effect of METTL3 knockdown on ccRCC cell migration and invasion (Fig. S9C and D).

Next, to investigate the specific mechanism by which METTL3 regulates TCF7L2, we employed actinomycin D treatment to impede novel RNA biosynthesis and subsequently assessed TCF7L2 mRNA levels. The results demonstrated that decreased METTL3 expression reduced the stability of TCF7L2 mRNA (Fig. 6H), whereas overexpression of METTL3 enhanced TCF7L2 stability (Fig. S9E). Further validation was performed through MeRIP assay, which verified that METTL3 knockdown diminished the modification levels of m^6^A in TCF7L2 mRNA (Fig. 6I). To explore the interaction between METTL3 and TCF7L2, we produced luciferase reporter plasmids comprising the wild-type and mutant sequences of TCF7L2 3′-UTR (Fig. 6J). The luciferase reporter assay results indicated that METTL3 knockdown suppressed TCF7L2 expression through the 3394 site within the TCF7L2 3′-UTR, rather than the 2643 site (Fig. 6K). These findings indicate that HIF2α regulates the m^6^A methylation levels of TCF7L2 through METTL3, resulting in the up-regulation of TCF7L2 expression.

After elucidating the role of METTL3 in stabilizing TCF7L2 mRNA through m^6^A modification, our focus shifts to unravel the underlying mechanisms driving METTL3’s stabilizing effect. In this context, we focus on m^6^A readers, essential components that mediate downstream effects. Conducting a correlation analysis between m^6^A readers and TCF7L2 in the TCGA-KIRC database, YTHDC1 emerges as a prominent candidate (Fig. S10A). Moreover, considering that both YTHDC1 and TCF7L2 are localized in the cell nucleus [53], YTHDC1 is likely to be involved in safeguarding the m^6^A-modified TCF7L2. YTHDC1 was either knocked down or overexpressed in ccRCC cells to validate its role in TCF7L2 mRNA stability. The results showed that YTHDC1 knockdown markedly inhibited the expression of TCF7L2 (Fig. S10B and C), while YTHDC1 overexpression markedly up-regulated TCF7L2 expression (Fig. S10D and E). RIP-qPCR was performed to confirm whether YTHDC1 serves as the reader for m^6^A methylation on TCF7L2 mRNA. The results indicated that YTHDC1 knockdown led to a reduction in the binding of TCF7L2 mRNA, while overexpression had the opposite effect (Fig. S10F). Additionally, the knockdown of METTL3 inhibited the binding of YTHDC1 to TCF7L2 mRNA (Fig. S10G). Furthermore, the knockdown of YTHDC1 resulted in decreased stability of TCF7L2 mRNA, while overexpression of METTL3 could reverse the reduced stability of TCF7L2 mediated by YTHDC1 knockdown (Fig. S10H and I). In summary, the stability of TCF7L2 mRNA is regulated through the METTL3–YTHDC1 axis in an m^6^A-dependent manner.

To investigate the specific regulatory mechanism between HIF2α and METTL3, we first considered transcriptional regulation. Based on the sequence information of the hypoxia-inducible element, 2 potential HIF2α binding sites, site 1 and site 2, were predicted in the 2,000-bp upstream promoter region of the METTL3 transcription start site (Fig. 6L). ChIP results demonstrated that HIF2α can bind to both site 1 and site 2 binding sites (Fig. 6M). Subsequently, we constructed truncated plasmids based on these 2 binding sites for luciferase reporter gene assays. The results confirmed that HIF2α mainly activates the transcription of TCF7L2 through binding to site 2 (Fig. 6N). Taken together, our findings suggest that HIF2α, through transcriptional activation of METTL3 expression, mediates the up-regulation of TCF7L2 expression by promoting its m^6^A methylation modification.

### TCF7L2, as a target gene of HIF2α, promotes ccRCC metastasis in vivo

**Fig. 7.**
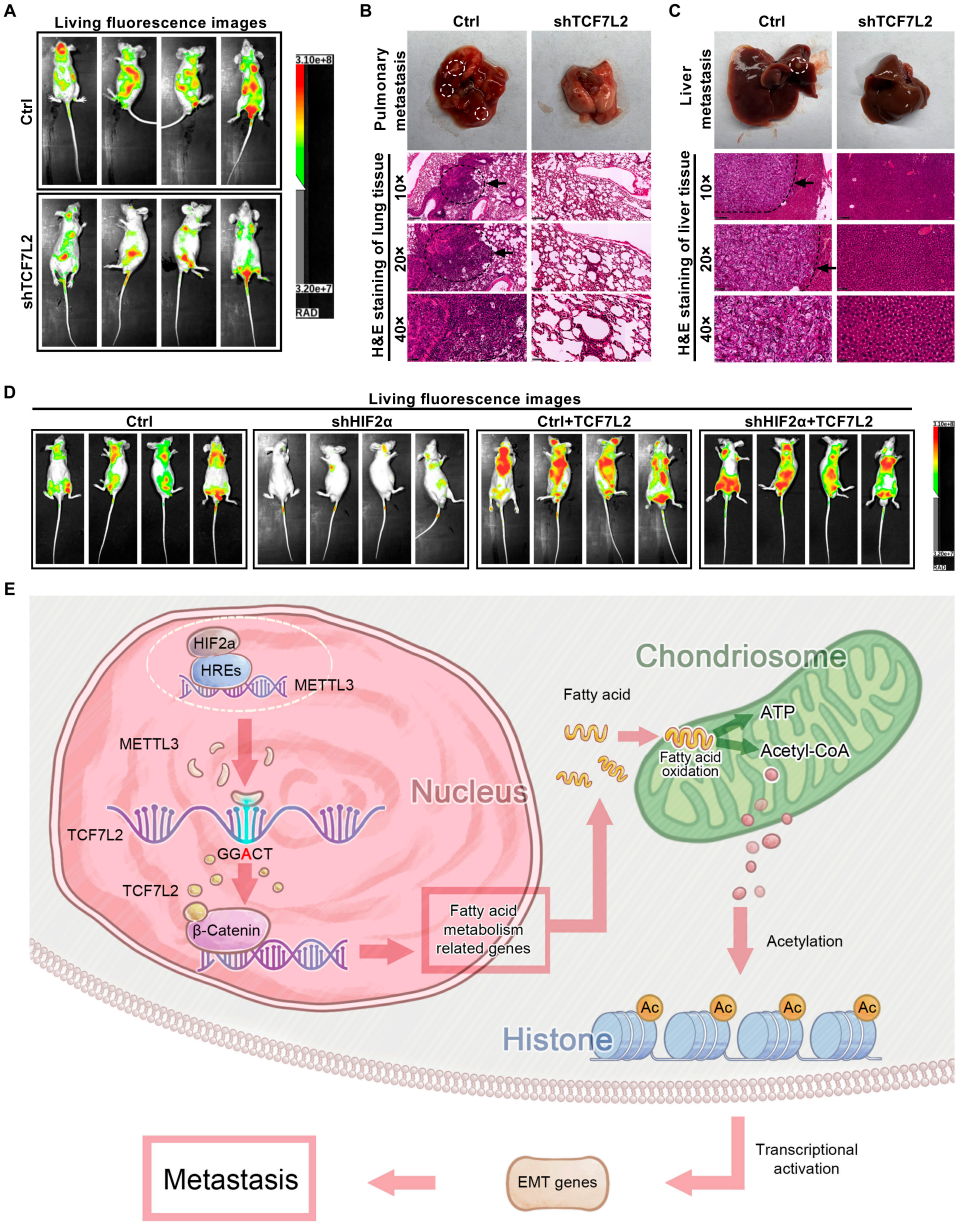
TCF7L2, as a target gene of HIF2α, promotes ccRCC metastasis in vivo. (A) Living fluorescence images of the metastasis model constructed with TCF7L2 knockdown and negative control A498 cells. (B) H&E staining of lung tissue from TCF7L2 knockdown group and negative control group nude mice. (C) H&E staining of hepatic tissue from TCF7L2 knockdown group and negative control group nude mice. (D) Living fluorescence images of the metastasis model constructed with functional recovery cells. (E) The proposed mechanistic model in this study.

To evaluate the TCF7L2 function in ccRCC metastasis in vivo and validate its regulation by HIF2α, we constructed a nude mouse model of ccRCC metastasis by intravenously introducing A498 cells expressing GFP fluorescence. In vivo fluorescence imaging experiments demonstrated that TCF7L2 depletion markedly suppressed ccRCC metastasis in live animals (Fig. 7A and Fig. S7A). Histological analysis of lung and liver tissues in the metastasis model, using hematoxylin and eosin (H&E) staining, indicated that TCF7L2 knockdown attenuated ccRCC metastasis in the lung and liver (Fig. 7B and C). Furthermore, a functional recovery metastasis model was established to validate the role of TCF7L2 as regulated by HIF2α in vivo. The findings exhibited that TCF7L2 overexpression reversed the suppressive impact of HIF2α knockdown on ccRCC metastasis (Fig. 7D and Fig. S7B). These findings indicate that TCF7L2 promotes the metastatic ability of ccRCC in vivo, and TCF7L2 is a crucial downstream target gene regulated by HIF2α in the context of ccRCC metastasis.

Based on these findings, we have put forth a novel conceptual framework elucidating the regulatory role of HIF2α in ccRCC metastasis. HIF2α interacts with HREs within the METTL3 promoter region to transcriptionally activate METTL3 expression. Elevated expression of METTL3 promotes enhanced m^6^A methylation modification of TCF7L2 mRNA, thereby stabilizing TCF7L2. Stabilized TCF7L2 forms a complex with β-catenin, stimulating the canonical Wnt signaling and promoting the transcription of downstream genes connected to de novo fatty acid formation. This leads to increased mitochondrial FAO. The elevated FAO generates acetyl-CoA, which functions as a substrate for histone acetylation, activating the transcription of EMT genes and facilitating ccRCC metastasis (Fig. 7E).

## Discussion

The Wnt pathway has a crucial function in diverse biological processes like cellular differentiation, proliferation, migration, and adhesion [54]. Abnormal Wnt signaling pathway activation has been connected to the development and advancement of several human cancers, including ccRCC [55]. Several Wnt signaling genes have been recognized as markers for ccRCC, and some of these genes have been demonstrated to regulate the progression of ccRCC [56–59]. The main oncogenic driver in ccRCC, HIF2α, exhibits abnormal activation, which is a crucial characteristic of ccRCC [8]. Hypoxia-induced stimulation of the Wnt mechanism has developed as a pivotal mechanism in driving tumor progression [60–62]. However, the specific mechanisms by which HIF2α participates in the Wnt pathway remain largely unexplored. In this investigation, we elucidated the HIF2α function in modulating canonical Wnt signaling in ccRCC via TCF7L2. Mechanistic investigations revealed that HIF2α transcriptionally activates METTL3, which mediates m^6^A modification of TCF7L2 mRNA. Up-regulation of METTL3 stabilizes TCF7L2 mRNA, resulting in Wnt signaling activation. Consequently, the expression of lipid synthesis genes is up-regulated, mitochondrial FAO increases, and acetyl-CoA is produced. This elevation in acetyl-CoA promotes histone acetylation and activates EMT, thus facilitating ccRCC metastasis.

The widespread RNA modification known as N6-methyladenosine (m^6^A) has a pivotal function in governing RNA fate by regulating RNA stability, notably by suppressing the cancer inhibitor gene expression [51,63]. Recent investigations have indicated that TCF7L2 mRNA is enriched with m^6^A modifications, and these modifications regulate the expression of TCF7L2 [64]. Consistent with this, our outcomes highlight the essential role of m^6^A modification in maintaining the stability of TCF7L2 mRNA and driving the activation of the canonical Wnt signaling in ccRCC. METTL3, serving as a vital component of the m^6^A methyltransferase complex, acts as the key “writer” accountable for catalyzing the m^6^A modification [65]. In cancer contexts, METTL3 has a critical function in modulating the target genes’ stability through an m^6^A-dependent mechanism. By depositing m^6^A modifications on mRNA molecules, METTL3 ensures the proper stability and integrity of these transcripts, ultimately influencing important cellular processes associated with cancer development and progression [66]. Importantly, hypoxia-induced up-regulation of METTL3 leads to increased m^6^A modification levels [67,68]. This provides a theoretical basis for investigating the regulatory mechanisms between HIF2α and METTL3. Moreover, emerging evidence suggests potential crosstalk between METTL3 and the Wnt pathway, with Wnt signaling dysregulation being associated with aberrant expression of METTL3 [69,70]. In this investigation, we determined METTL3 as an intermediate mediator of HIF2α in regulating TCF7L2. METTL3-mediated m^6^A modification in the 3′-UTR region contributes to TCF7L2 mRNA stabilization and canonical Wnt signaling mechanism activation by facilitating the canonical Wnt signaling target gene expression. Certainly, we cannot definitively establish that METTL3 is the sole intermediary through which HIF2α regulates TCF7L2. In our study, we observed that HIF2α can modulate the mRNA levels of TCF7L2, but it does not directly transcriptionally activate TCF7L2. Although we have ruled out the impact of pre-RNA modifications, we are still uncertain about the existence of other regulatory mechanisms.

Lipid metabolism reprogramming is a key factor in ccRCC progression. Up-regulation of key enzymes in the de novo synthesis pathway enhances FA formation, and excessive FAs are connected to TGs and stored as lipid droplets (LDs) for energy storage, which can be mobilized through FAO to generate ATP [71,72]. Lipidomic studies have revealed an increased utilization of FAs in ccRCC [73]. Previously, we reported that ccRCC cells deplete lipid storage through lipid browning mediated by uncoupling protein 1 (UCP1) [74]. In this study, we identify TCF7L2 as a major driver of lipid-based metabolic reprogramming in ccRCC, supporting cancer cell invasion potential through the activation of EMT. While some investigations have determined the lipids’ role in EMT [36,75], our investigation addresses this issue by integrating lipid synthesis and FAO. We demonstrate that the HIF2α-guided metabolic shift in lipid metabolism depends on TCF7L2, and the increased lipid synthesis supports the invasive capacity of ccRCC. Interestingly, although not demonstrated in our study, we observed an up-regulation of PLIN2 (involved in LD stability) expression in conjunction with TCF7L2. The potential implications of this finding in activating EMT warrant further investigation. Additionally, our evidence indicates that the production of acetyl-CoA leads to epigenetic regulation of EMT target genes through histone acetylation. Histone acetylation is a modification that adds acetyl groups to lysine residues on histones, modulating chromatin structure and gene expression [76]. Acetylated histones promote an open chromatin conformation, facilitating transcriptional activation of genes [77]. The acetyl groups required for histone acetylation are derived from the product of FAO, acetyl-CoA. These findings provide evidence that lipid metabolism reprogramming contributes to controlling cellular state transitions and offer a new explanation for the interplay between lipid metabolism and metastasis in ccRCC.

HIF2α, as the key oncogene in ccRCC, has been widely investigated in the occurrence and treatment of ccRCC. HIF2α activation regulates a diverse array of genes associated with the progression of ccRCC. Current research predominantly focuses on elucidating its functions in angiogenesis and cell cycle regulation [78]. In recent years, treatment options for metastatic ccRCC have made progress, including various targeted approaches and immunotherapies. Targeting HIF2α therapy has gained considerable attention, and both PT2385117 and PT2977 (recently identified as “MK-6482”) have demonstrated targeted anti-tumor activity in mouse xenograft models of ccRCC as HIF2α inhibitors [79–81]. However, notable adverse reactions remain an inevitable concern despite the ability of HIF2α inhibitors to inhibit ccRCC progression. Anemia and hypoxia are common adverse events associated with HIF2α inhibitors, with anemic patients requiring exogenous erythropoietin therapy and patients with respiratory distress requiring oxygen therapy [82]. These issues raise concerns about the pro-tumor metastasis role of HIF2α. In this study, we confirm that TCF7L2 is a key downstream mediator of HIF2α in regulating ccRCC metastasis and is linked to lipid metabolism reprogramming. These findings emphasize the importance of targeting the TCF7L2-mediated pathway as a potential treatment for the management of metastatic ccRCC. Our research findings support the use of targeted delivery strategies [83] within the context of the Wnt signaling to inhibit TCF7L2 expression as a potential therapeutic approach for treating ccRCC.

Our investigation indicates the previously unrecognized function of HIF2α in mediating the TCF7L2 mRNA stability through METTL3 in a manner based on m^6^A, regulating ccRCC fatty acid synthesis and EMT activation. It provides new insights into the HIF2α role in regulating ccRCC metastasis. Additionally, we identify the regulatory ccRCC lipid metabolism reprogramming effect on EMT. These findings offer opportunities for developing novel drugs targeting TCF7L2 and combination therapies involving lipid-lowering agents and existing targeted treatments, aiming to enhance the treatment results for individuals with advanced and metastatic ccRCC.

## Materials and Methods

### Specimens and cell lines of human ccRCC tissue

Human ccRCC tissue samples were acquired from the Urology Department at Wuhan Union Hospital (Hubei, China) with the appropriate authorization from the Institutional Review Board of Huazhong University of Science and Technology. Previous informed consent was acquired from the participants involved in the study. The tumor tissues were collected during radical nephrectomy and partial nephrectomy procedures and promptly preserved by snap-freezing in liquid nitrogen for subsequent utilization.

HK2 and HEK293T cell lines, as well as human ccRCC cell lines (A498, 786-O, CAKI-1, and OSRC), were procured from the American Type Culture Collection (Manassas, USA). The cells were cultured in high-glucose Dulbecco’s Modified Eagle’s Medium (DMEM) (Gibco, Wilmington, USA), treated with fetal bovine serum and 1% penicillin–streptomycin, and kept with 5% CO_2_ at 37 °C.

### RNA extraction and RT-qPCR

Cell/Tissue Total RNA Isolation Kit V2 (RC112-01, Vazyme, Nanjing, China) was utilized to isolate the Total RNA, based on the manufacturer’s directions. Subsequently, the HiScript III 1st Strand cDNA Synthesis Kit (R312-01, Vazyme, Nanjing, China) was employed to synthesize cDNA. To establish a standard curve and calculate the relative target concentration (qTOWER, Analytik Jena, Jena, Germany), the Sybr Green Master Mix (#11203ES03, Yeasen, Shanghai, China) was utilized to amplify the diluted cDNA samples. The relative expression levels of RNA among different groups were evaluated using the 2^−ΔΔCt^ method. The sequences of the primer employed in this investigation are illustrated in Table S1.

### Protein extraction and Western blot

Protein lysates were produced by adding protease suppressor cocktail (P8340, Sigma-Aldrich, Missouri, USA) and phenylmethylsulfonyl fluoride (PMSF) protease suppressor (36978, Thermo Fisher Scientific, Massachusetts, USA) to radio-immunoprecipitation assay (RIPA) buffer (P0013B, Beyotime, Wuhan, China). The BCA Protein Assay Kit (23225, Thermo Fisher Scientific, Massachusetts, USA) was employed to identify protein levels. Denatured proteins (approximately 30 to 50 μg) were then transferred onto a polyvinylidene fluoride (PVDF) membrane using sodium dodecyl sulfate-polyacrylamide gel electrophoresis (SDS-PAGE). The antibodies employed in this trial are presented in Table S2. Immunoblotting was conducted using the Molecular Imager ChemiDox XRS+ imaging system and examined utilizing the Image Lab program (Bio-Rad Laboratories). Original blots of the Western blotting are presented in File S2.

### Cell infection and transfection

Lipofectamine 3000 Transfection Reagent (L3000001, Invitrogen, Massachusetts, USA) was utilized to transfect the Custom siRNAs (Table S3) obtained from Genepharma (Shanghai, China) into cells. Lentiviruses containing short hairpin RNA (shRNA) targeting HIF2α and TCF7L2 (Supplementary Table S3) and lentivirus for TCF7L2 overexpression (vector: Ubi-MCS-3FLAG-CBh-gcGFP-IRES-puromycin) were acquired from Genechem (Shanghai, China). The lentiviruses were utilized to subsequently infect A498 and 786-O cells depending on the manufacturer’s directions. To construct the overexpression plasmid, human methyltransferase-like-3 (METTL3) was cloned into the GM19315-lenti-CMV-MCS-PGK-Puro vector (Genomeditech, Shanghai, China). Cell lysates and total RNA were obtained 72 h post transfection or infection, and Western blotting and qPCR analysis were employed to confirm the efficiency of METTL3 overexpression.

### Immunohistochemistry

Tissue sections underwent deparaffinization and heat-induced antigen retrieval using 10 mM sodium citrate buffer (pH 6.0) to enhance antigen detection. Endogenous peroxidase activity was blocked. Then, the segments were subjected to incubation with primary antibodies against cleaved caspase-3 (#9664, Cell Signaling Technology), Ki-67 (#9449, Cell Signaling Technology), and TCF7L2. Following antigen retrieval, peroxidase-conjugated secondary antibodies were applied, and the staining was visualized using diaminobenzidine. Image acquisition was performed using an optical microscope, namely, the #DSZ2000 model manufactured by UOP Photoelectric Technology located in Chongqing, China.

### Immunofluorescence

The cells were seeded onto circular coverslips at a density of 10^5^ cells per coverslip (Biosharp, Hefei, Anhui, China). After multiple washes with phosphate buffered saline (PBS), the cells were treated for 10 min with 4% paraformaldehyde, permeabilized for 10 min with 0.5% Triton X-100, and blocked with bovine serum albumin of 5%. Following this, the slides were subjected to overnight incubation at a temperature of 4 °C with the primary antibody. Subsequently, a 2-h incubation at room temperature was conducted using Alexa Fluor 594-conjugated goat anti-rabbit immunoglobulin G (IgG; #AS039, diluted 1:250, ABclonal, Wuhan, Hubei, China). At room temperature, DAPI solution (#C1002, Beyotime) was used for nuclear staining for 10 min. A DMI3000B fluorescence microscope (Leica, Wetzlar, Hessen, Germany) was utilized to capture fluorescent images.

### Cell viability assay

A 96-well plate was utilized for seeding cells with a density of 2,000 cells per well. The determination of the cell growth rate was conducted employing the Cell Counting Kit-8 (A311-02CCK8, Vazyme, Nanjing, China) based on the directions of the manufacturer. After removing the culture medium from the wells, the CCK8 solution was mixed with DMEM at a ratio of 1:10. Consequently, 110 μl of the mixture was supplemented to both the blank wells and the test wells. The plate was subjected to incubation in a water bath set at a temperature of 37 °C, while being kept in a dark environment. The absorbance at 450 nm was identified utilizing a spectrophotometer (NanoDrop Technologies, Wilmington, DE, USA) at 0, 24, 48, 72, and 96 h.

### Transwell assay

The experimental procedures were conducted as described previously [84]. Matrigel (diluted 1:8, #356234, Corning, NY, USA) was applied to the superior chamber of Transwell inserts (#REF3422, Corning, NY, USA). At a density of 8×10^5^ cells per well, the cells were introduced into the upper chamber. After 1 day, the cells in the inferior chamber were treated with methanol for 10 min. Subsequently, staining was performed using a 0.05% crystal violet solution for 10 min, then subjected to 3 rinses with PBS. An optical microscope (#DSZ2000, UOP Photoelectric Technology, Chongqing, China) was employed to capture the images. The ability of cells to migrate was evaluated utilizing Transwell inserts without Matrigel.

### Wound healing assay

Cells were cultivated in a 6-well plate until they achieved a confluency of 80%. The serum-free culture medium was then replaced, and the cells were subjected to 8 h of incubation. To create cell scratches, a 10-μl pipette tip was used. Afterward, the cells were rinsed thoroughly with PBS to eliminate any remaining debris. Cell images were obtained using an optical microscope (#DSZ2000, UOP Photoelectric Technology, Chongqing, China) at 0, 12, and 24 h as the designated time points for capturing photographs.

### Cell oil red O staining

Typically, 10^5^ cells underwent seeding in a 6-well plate. Oil Red dye was prepared by mixing saturated Oil Red solution (C0157M, Beyotime, Wuhan, China) with ultrapure water in a 2:3 ratio. Cells were treated for 10 min with 4% paraformaldehyde and then air-dried in a 6-well plate. Subsequently, at room temperature, the prepared Oil Red dye was supplemented, and the cells were stained for 1 h and protected from light. Then, PBS was utilized to remove excessive Oil Red dye, and the plate was air-dried before capturing images using an optical microscope (#DSZ2000, UOP Photoelectric Technology, Chongqing, China).

### Measurement of TG content and TCH content

The cells were seeded onto 10-cm cell culture plates and subjected to a 24-h incubation period. Subsequently, the cells were collected and 0.9 ml of Triton X-100 (#P0096, Beyotime, Wuhan, China) was added. Subsequently, the levels of TG and TCH were detected in the supernatant following the instructions provided with the TG Detection Kit (#A110-1-1, Jiangsu Nanjing Jiancheng, China) and the TCH Detection Kit (#A111-1-1, Jiangsu Nanjing Jiancheng, China).

### Measurement of OCR

The cells were cultured in DMEM for 24 h to achieve a fusion efficiency of 60%. Afterward, the cells were switched to FAO detection medium and continued to incubate for 45 min at 37 °C. Subsequently, after the incubation, the culture dishes were taken out, and 30 μl of palmitic acid-BSA substrate or control BSA, along with 15 μl of recombinant MitoXpress reagent, was added to the wells. MitoXpress Xtra Oxygen Consumption Assay kit (Luxcel Bioscience, Cork, Ireland) was employed to detect the OCR, and Envision Multimode Plate Reader (Perkin Elmer, Massachusetts, USA) was utilized for the quantification.

### Cellular ATP measurement

The cells were distributed in a 96-well plate with a seeding density of 10,000 cells per well. After incubation for the desired time period, the culture medium was aspirated, and 100 μl of CellTiter-Glo 2.0 reagent (PRG9242, Promega, Wisconsin, USA) was supplemented to each well. Cell lysis and subsequent release of intracellular ATP were induced by gently mixing the plate on an orbital shaker for a duration of 2 min. A luminometer (Perkin Elmer, Massachusetts, USA) was utilized to detect the luminescence after a 10-min incubation at room temperature to stabilize the luminescent signal. The luminescent signal intensity was directly relative to the ATP content in the cells and was used as an indicator of cellular ATP levels.

### RNA stability assay

The cells were cultivated in 6-well plates and left to achieve a level of 50% confluence. Subsequently, the cells were supplemented with 5 μg/ml cycloheximide (Sigma-Aldrich). At 0, 3, 6, 9, and 12 h after treatment, cells were collected, and RNA was obtained utilizing an RNA extraction kit following the manufacturer’s directions. To detect mRNA decay rates, the extracted RNA was then reversely transcribed and subjected to quantitative real-time PCR (RT-qPCR). The mRNA levels were standardized to a reference gene, and appropriate analysis methods were employed to calculate the relative mRNA decay rates.

### TOP/FOP flash luciferase reporter assay

The TOP/FOP-flash luciferase reporter assay was conducted following the designated protocol. Briefly, TOP/FOP-flash reporter gene and pTK-RL plasmid were utilized to co-transfect the cells before being subjected to their respective treatments. To stimulate the canonical Wnt signaling mechanism, Wnt3a was added at a concentration of 20 ng/ml. The Dual-Luciferase Reporter Assay System from Promega was employed to detect the luciferase activities of firefly and Renilla. The activity of the TOP/FOP-Flash reporter gene was determined by calculating the relative proportion of firefly luciferase to Renilla luciferase activities.

### Luciferase reporter assay

Wild-type sequences containing the m^6^A site of TCF7L2, mutant sequences with point mutations at the m^6^A site of TCF7L2, and truncated and full-length sequences of the METTL3 promoter region were synthesized and cloned into the XhoI location of the GV354 vector (Genechem, Shanghai, China). HEK293T, A498, or 786-O cell lines were cultured in 12-well plates and incubated until they achieved 60% confluence during a 24-h period. The reporter plasmid (100 ng) was co-transfected with specific siRNA or overexpression plasmids using Lipofectamine 3000. After 48 h, a luminescent substrate for firefly and Renilla luciferase was added, and a luminometer (Promega, Wisconsin, USA) was utilized to detect the luciferase activities. The relative luciferase activity was estimated by standardizing the firefly luciferase intensity to the Renilla luciferase intensity.

### MeRIP-qPCR

The Magna Methylated RNA Immunoprecipitation m^6^A Kit (Millipore, Massachusetts, USA) was employed to investigate the m^6^A modification of specific genes based on the manufacturer’s guidelines. A total of 5 μg of anti-m^6^A antibody (CS220007, Millipore, Massachusetts, USA) or regular mouse IgG (CS200621, Millipore, Massachusetts, USA) was subjected to a pre-washing step and then treated at room temperature with Magna ChIP protein A/G magnetic beads (CS203152, Millipore, Massachusetts, USA) for a duration of 1 h. Next, the complexes formed by the antibodies and beads were mixed with pure poly-(A) RNA and subjected to RT-qPCR analysis to examine the enrichment of m^6^A-modified mRNA. The relative m^6^A enrichment in each specimen was determined by normalizing to the input. The sequences of the primer utilized for MeRIP-qPCR are provided in Table S1.

### Chromatin immunoprecipitation

The ChIP assay was conducted using the SimpleChIP Kit (Agarose Beads) (CST, 22188S, Boston, USA) as per the directions of the manufacturer. Rabbit anti-HIF2α antibody (#59973S, CST, Boston, USA) or normal rabbit IgG (#2729, CST, Boston, USA) were utilized to conduct immunoprecipitation. The recovered DNA was then subjected to qPCR amplification to assess the HIF2α binding to the promoter location of METTL3. The relative enrichment was normalized to the IgG control. The sequences of primer utilized are provided in Table S1.

### In vivo metastasis assay

Male BALB/c nude mice, aged 6 weeks, were acquired from Vital River Company located in Beijing, China. These mice were kept in an environment that was free from any particular pathogens. All animal experiments were conducted following the guidelines and regulations authorized by the Institutional Animal Ethics Committee of Tongji Medical College (approval no. S1892).

To create a metastatic tumor model, 5×10^6^ A498 cells were intravenously administrated into the vein of nude mice tail (*n* = 6) to assess the metastatic potential of the tumor cells. After a period of 6 weeks, in vivo fluorescence imaging of the nude mice was conducted using the LagoX system (Spectral Instruments Imaging, Tucson, AZ, USA). After undergoing imaging procedures, the mice were euthanized in a humane manner, and their lungs and livers were then obtained for the purpose of conducting H&E staining.

### RNA sequencing

The Cell/Tissue Total RNA Isolation Kit V2 (RC112-01, Vazyme, Nanjing, China) was employed to obtain RNA specimens from treated and control cells. The isolated RNA samples were then subjected to mRNA purification and quantification. The mRNA molecules were fragmented using divalent cations and subsequently used for PCR amplification. The resulting PCR products were used to construct libraries for sequencing. Paired-end sequencing was conducted on the system of Illumina HiSeq 4000 (Illumina, San Diego, CA, USA) following the manufacturer’s recommendations. To guarantee the robustness and reproducibility of the results, each experimental group was composed of 3 biological replicates and 3 technical repetitions.

For data analysis, the GO (GO, http://www.geneontology.org/) and Kyoto Encyclopedia of Genes and Genomes (KEGG, http://www.genome.jp/kegg) databases were utilized to identify pathways enriched with statistically significant genes. The analysis aimed to uncover functional insights and molecular pathways associated with the experimental conditions. The technical and methodological aspects of whole transcriptome sequencing were supplied by Majorbio Biotechnology Co., Ltd. (Shanghai, China), ensuring expertise and quality in the sequencing process.

### Bioinformatics analysis

Publicly accessible RNA-seq information was obtained from various databases, like the Cancer Genome Atlas (TCGA) (https://portal.gdc.cancer.gov), Gene Expression Omnibus (GEO) (GSE213324, https://www.ncbi.nlm.nih.gov/gds), and GENT2 (http://gent2.appex.kr/gent2/) databases. Pearson correlation coefficient analysis was conducted to assess the correlation between HIF2α and TCF7L2 at the mRNA level. The statistical significance was determined at a significance level of *P* < 0.05.

Differential expression genes (DEGs) were determined utilizing the “DESeq2” package, and Sankey diagrams were generated employing the “networkD3” package in the R program environment (R Core Team, https://www.r-project.org). DEGs were defined based on adjusted *P* < 0.05 and |log fold-change| > 2 criteria.

In order to get a deeper understanding of the biological activities and pathways associated with the DEGs, a functional enrichment analysis was performed. This analysis involved GSEA, GO, and KEGG analysis.

To explore the probable m^6^A modification region in the sequence of TCF7L2 cDNA, the SRAMP (http://www.cuilab.cn/sramp) was utilized.

### Statistical analysis

Statistical analysis was performed utilizing Excel 2021 (Microsoft, Redmond, WA, USA) and SPSS 26.0 (IBM, Armonk, NY, USA). The data are expressed as mean ± standard deviation. Depending on the specific experimental design, various statistical tests were employed, including 2-tailed unpaired or paired *t* tests, analysis of variance (ANOVA), non-linear regression analysis, and Pearson correlation coefficient. A significance level of *P* < 0.05 was considered statistically significant.

#### Ethics approval and consent to participate

The study protocol was approved by the ethics committee of Huazhong University of Science and Technology (permit number: 2021IEC072). The tissue samples were obtained with written informed consent from each patient. The animal study was carried out in compliance with the guidance suggestion of Hubei Provincial Experimental Animal Research Centre (Certificate number: 2019S1892).

## Data Availability

The data supporting the conclusions of this investigation may be acquired from the corresponding author upon an appropriate demand. The sequencing datasets have been submitted in a suggested data repository, namely, the Science Data Bank (http://www.scidb.cn/). The sequencing datasets may be accessed through the private URL provided at https://www.scidb.cn/en/s/Nz2qEv.
