# Supplementary figures and images for: HIF2α Promotes Cancer Metastasis through TCF7L2-Dependent Fatty Acid Synthesis in ccRCC
Source: Research (Wash D C). 2024 Feb 22;7:0322. doi: 10.34133/research.0322 (PMC10882601; doi:10.34133/research.0322)

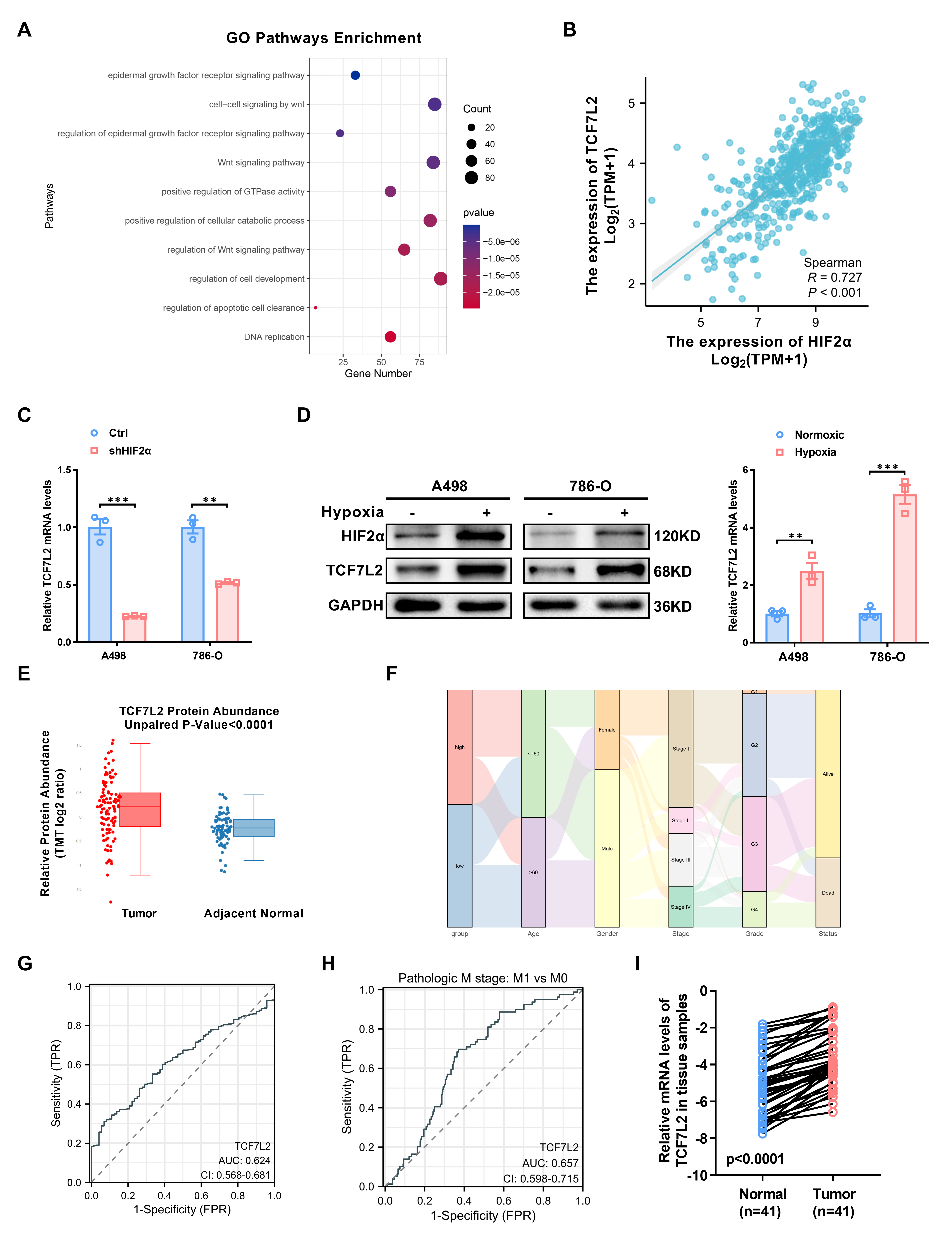

Supplement: Supplementary 1 — Figs. S1 to S11 [file research.0322.f1.zip › Figure S1.tif]

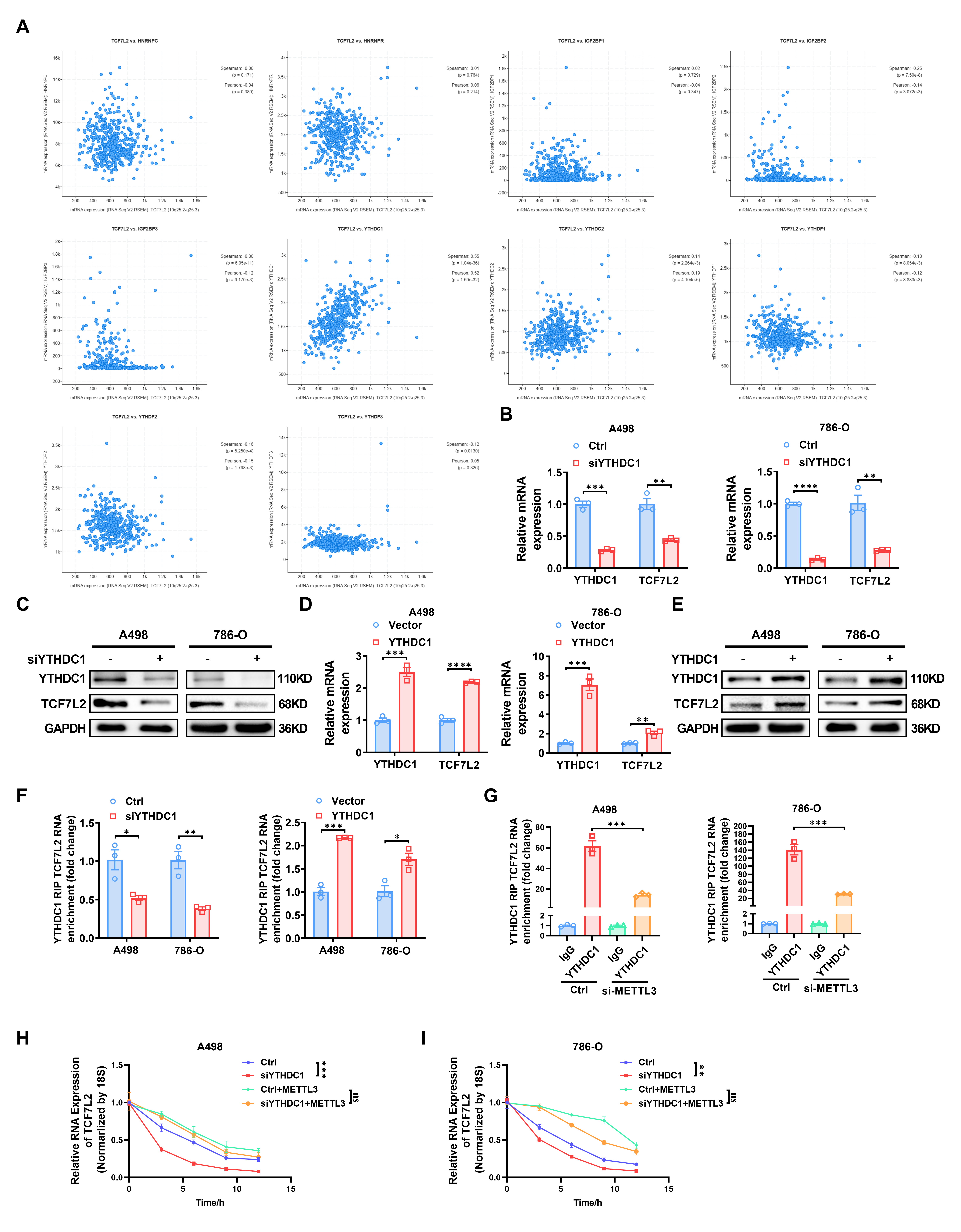

Supplement: Supplementary 1 — Figs. S1 to S11 [file research.0322.f1.zip › Figure S10.tif]

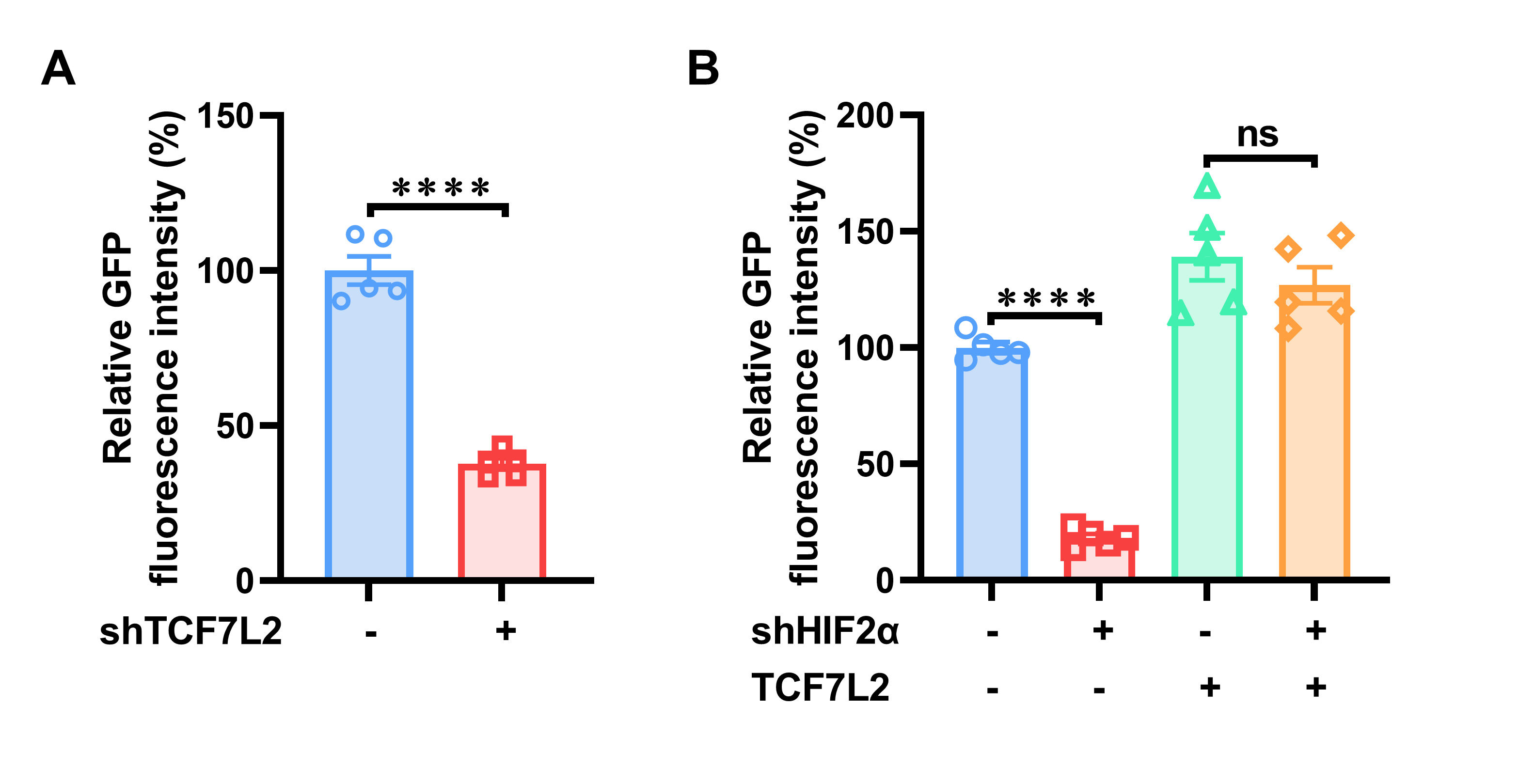

Supplement: Supplementary 1 — Figs. S1 to S11 [file research.0322.f1.zip › Figure S11.tif]

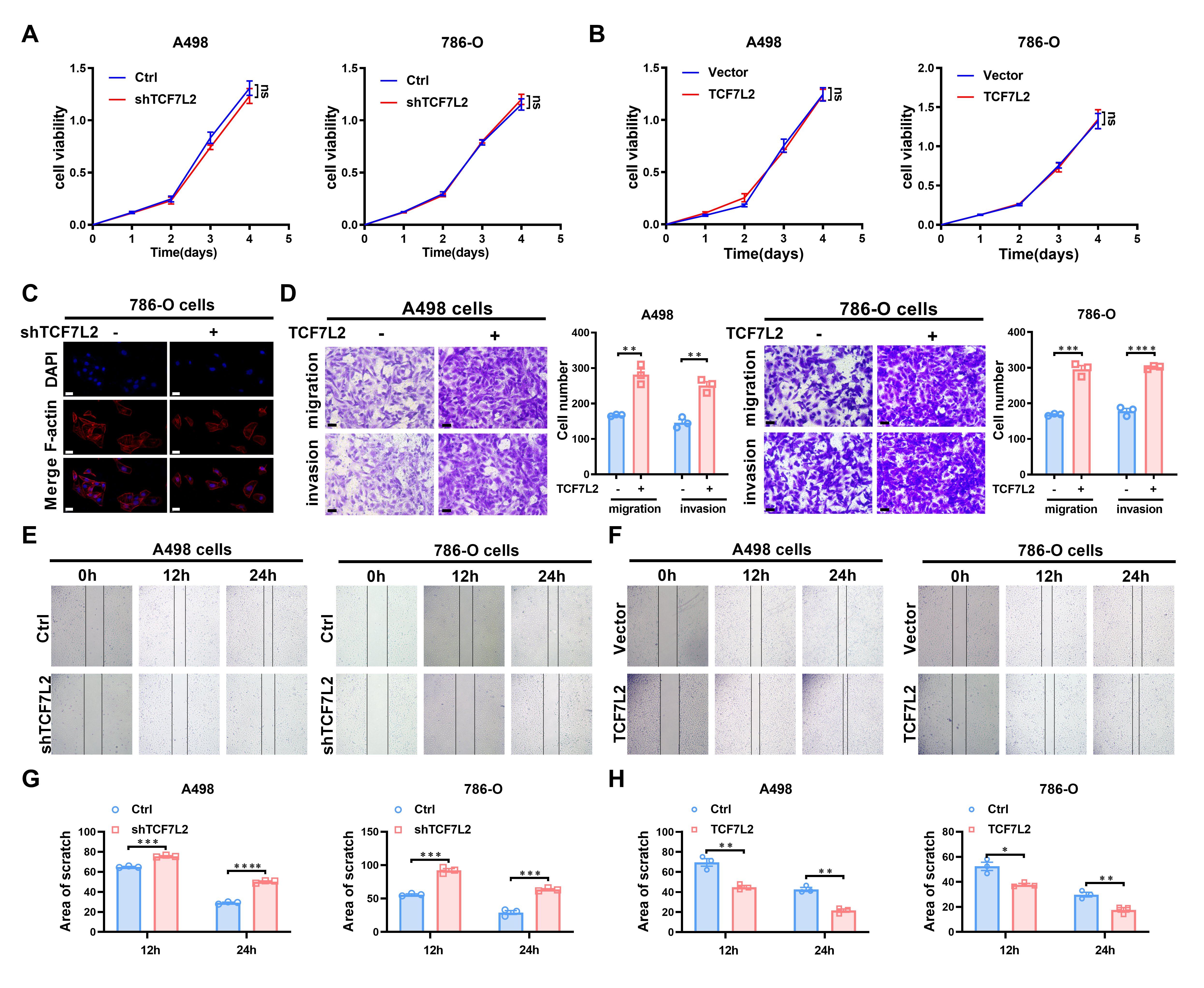

Supplement: Supplementary 1 — Figs. S1 to S11 [file research.0322.f1.zip › Figure S2.tif]

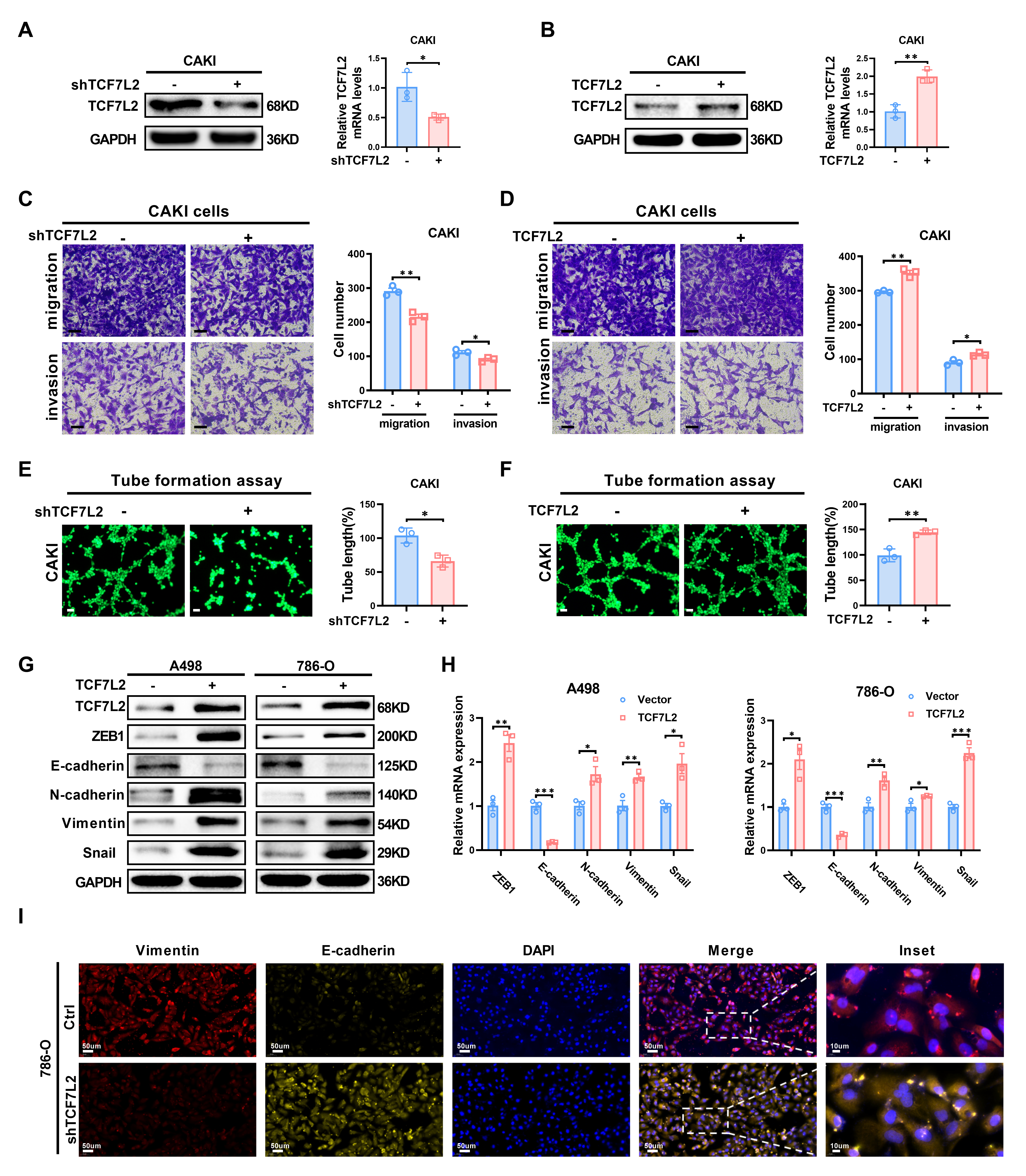

Supplement: Supplementary 1 — Figs. S1 to S11 [file research.0322.f1.zip › Figure S3.tif]

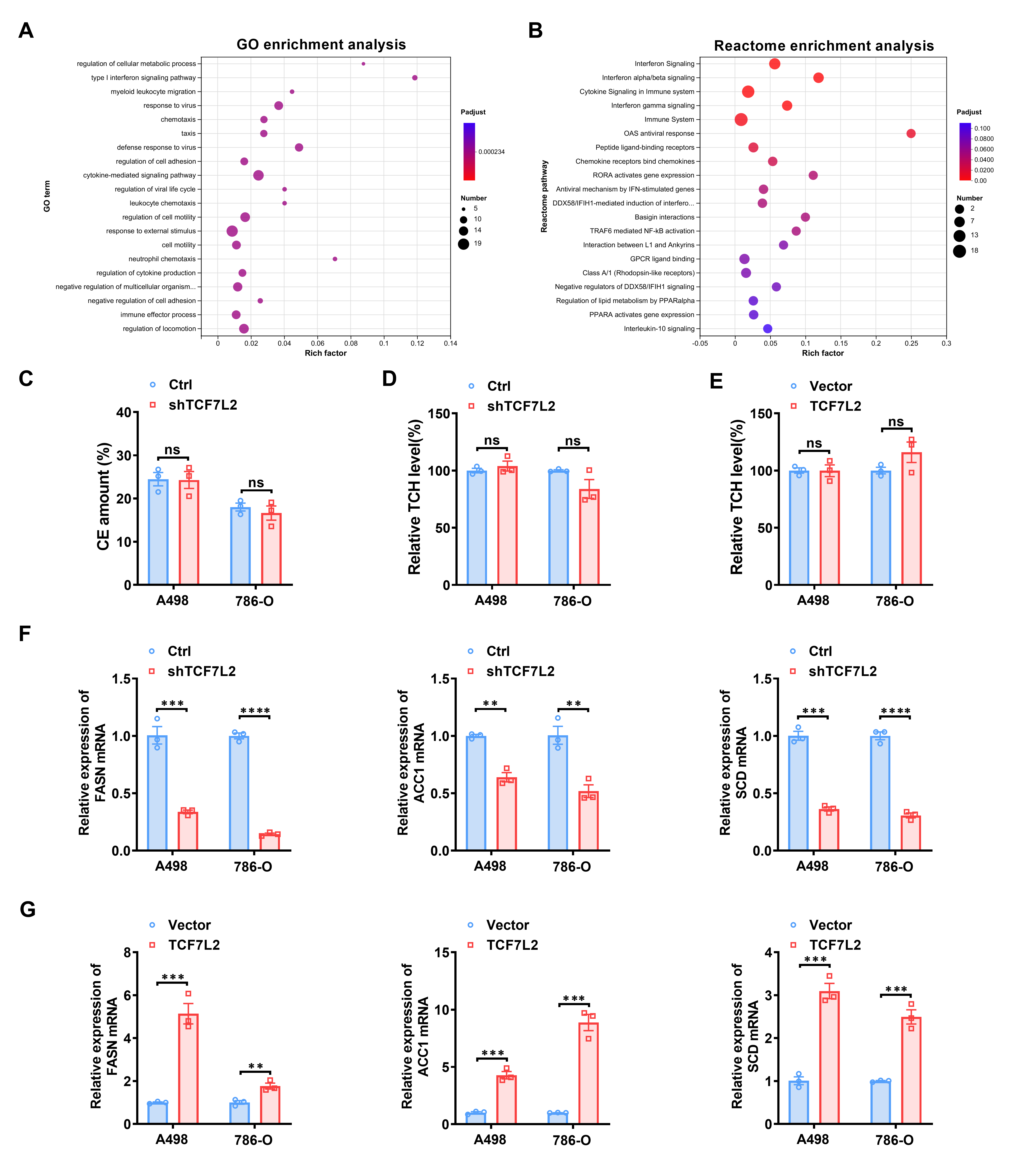

Supplement: Supplementary 1 — Figs. S1 to S11 [file research.0322.f1.zip › Figure S4.tif]

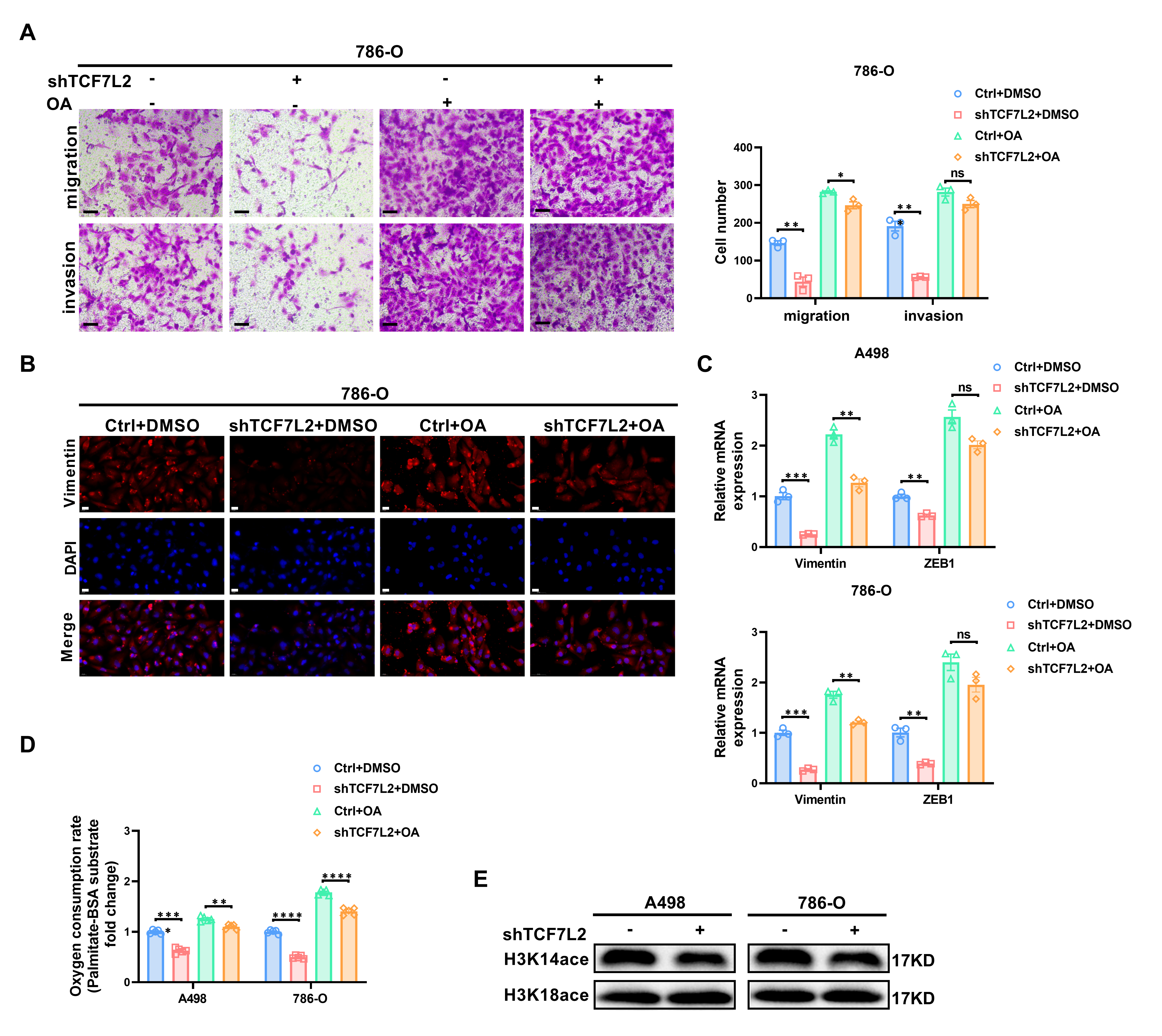

Supplement: Supplementary 1 — Figs. S1 to S11 [file research.0322.f1.zip › Figure S5.tif]

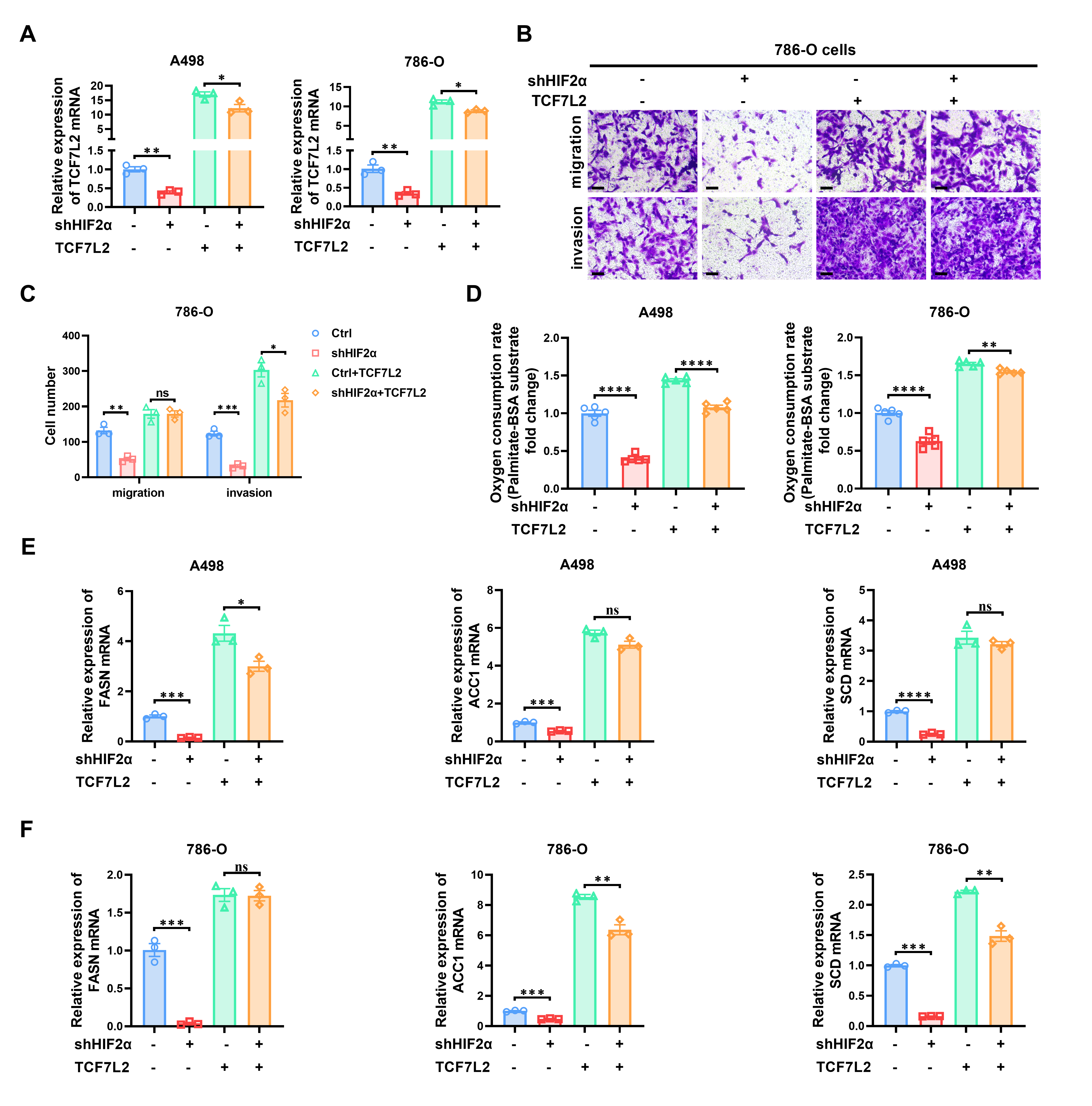

Supplement: Supplementary 1 — Figs. S1 to S11 [file research.0322.f1.zip › Figure S6.tif]

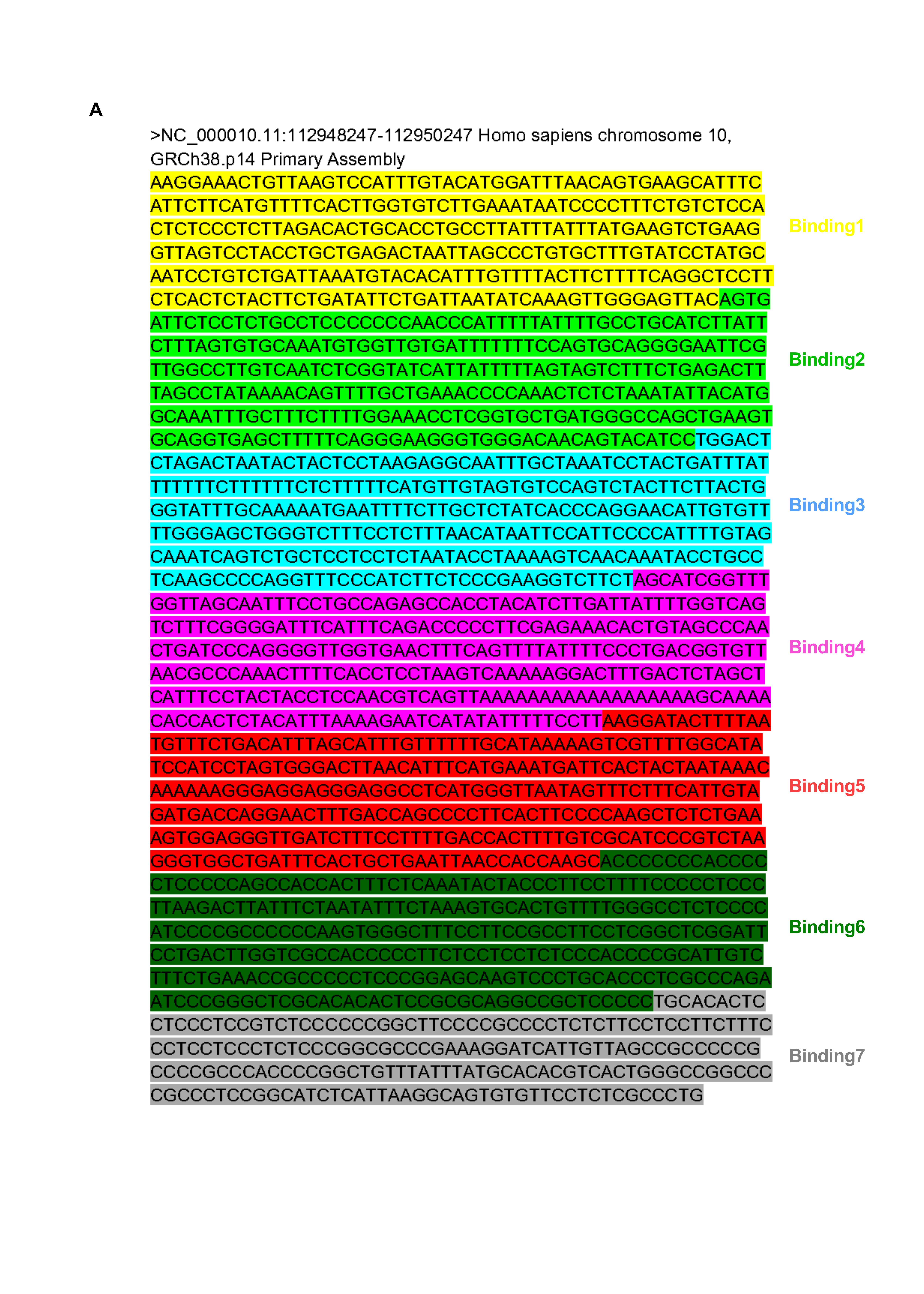

Supplement: Supplementary 1 — Figs. S1 to S11 [file research.0322.f1.zip › Figure S7.tif]

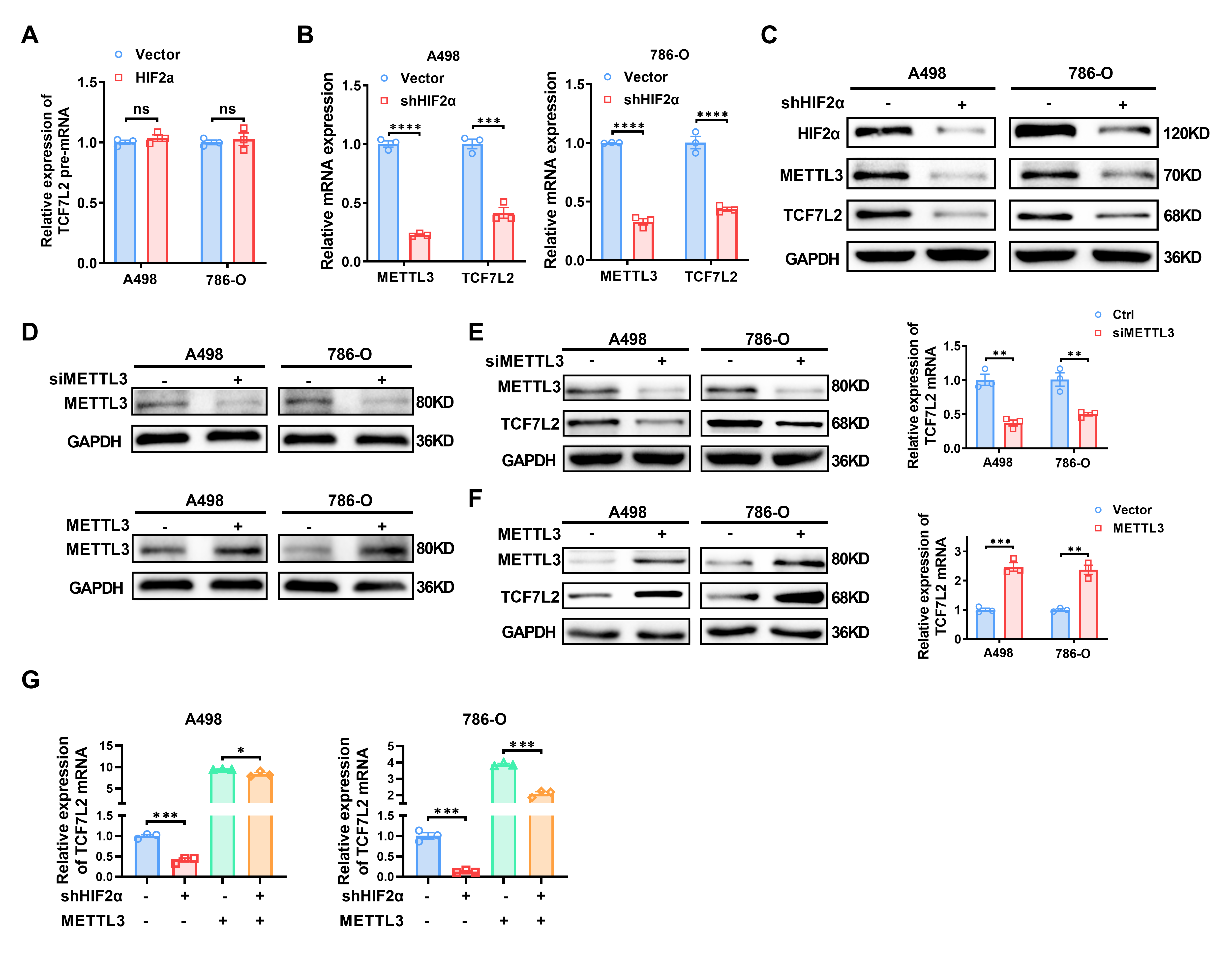

Supplement: Supplementary 1 — Figs. S1 to S11 [file research.0322.f1.zip › Figure S8.tif]

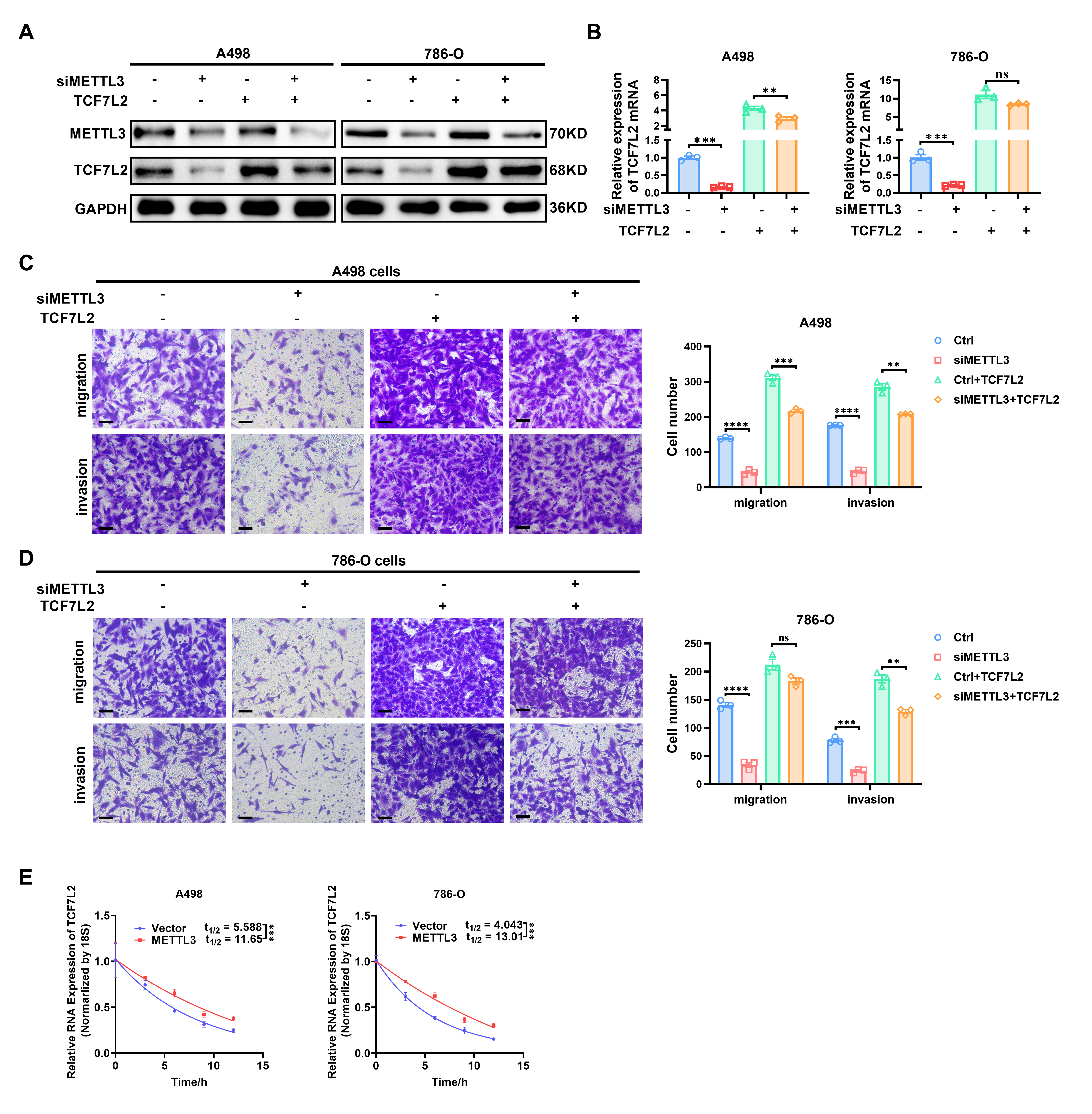

Supplement: Supplementary 1 — Figs. S1 to S11 [file research.0322.f1.zip › Figure S9.tif]
