# Supplementary material for: HIF2α Promotes Cancer Metastasis through TCF7L2-Dependent Fatty Acid Synthesis in ccRCC
Source: Research (Wash D C). 2024 Feb 22;7:0322. doi: 10.34133/research.0322 (PMC10882601; doi:10.34133/research.0322)
Supplement: Supplementary 2 — Files S1 to S3 [file research.0322.f2.zip › Supplemental file 1.docx]

**Supplementary Table S1** Sequences of primers of PCR, qPCR, and MeRIP-qPCR

| Gene | | Sequences (5'- -3') |
| --- | --- | --- |
| TCF7L2 | Forward | TGGAGGGCTCTTTAAGGGG |
|  | Reverse | GATCCGTTGGGGAGGTAGG |
| GAPDH | Forward | CCAGAACATCATCCCTGCCT |
|  | Reverse | CCTGCTTCACCACCTTCTTG |
| HIF2a | Forward | ACAGGTGGAGCTAACAGGAC |
|  | Reverse | CCGTGCACTTCATCCTCATG |
| ZEB1 | Forward | AGGGCACACCAGAAGCCAG |
|  | Reverse | GAGGTAAAGCGTTTATAGCCTCTATCA |
| E-cadherin | Forward | CCCGGGACAACGTTTATTAC |
|  | Reverse | GCTGGCTCAAGTCAAAGTCC |
| N-cadherin | Forward | CCTCCAGAGTTTACTGCCATGAC |
|  | Reverse | GTAGGATCTCCGCCACTGATTC |
| Vimentin | Forward | TACAGGAAGCTGCTGGAAGG |
|  | Reverse | ACCAGAGGGAGTGAATCCAG |
| Snail | Forward | ccccaatcggaagcctaact |
|  | Reverse | gacagagtcccagatgagca |
| FASN | Forward | ccctcatctccccactcatc |
|  | Reverse | cagcgtcttccacactatgc |
| ACC1 | Forward | caactttgtgcccacggtta |
|  | Reverse | tttgtcaggaagaggcggat |
| SCD | Forward | tgcccaccacaagttttcag |
|  | Reverse | catcagcaagccaggtttgt |
| METTL3 | Reverse | CTTGCATGGATTCTGAGGCC |
|  | Forward | GTCAGCCATCACAACTGCAA |
| 3'UTR of TCF7L2 | Forward | ccatgaatgcagtgccgtta |
|  | Reverse | gggacgacatacaggtacga |
| 5'UTR of TCF7L2 | Forward | cacgagcacctcctgtatct |
|  | Reverse | gaaaagagggaggaggggag |
| CDS of TCF7L2 | Forward | catcatgatccccgacctga |
|  | Reverse | tagagacaatgtgtgccggt |
| ChIP-Binding1 | Forward | TCCCTCTTAGACACTGCACC |
|  | Reverse | AGAGTGAGAAGGAGCCTGAA |
| ChIP-Binding2 | Forward | GGAATTCGTTGGCCTTGTCA |
|  | Reverse | TACTGTTGTCCCACCCTTCC |
| ChIP-Binding3 | Forward | TTCATGTTGTAGTGTCCAGTCT |
|  | Reverse | AGAGGAGGAGCAGACTGATT |
| ChIP-Binding4 | Forward | GCCAGAGCCACCTACATCTT |
|  | Reverse | GAGGTGAAAAGTTTGGGCGT |
| ChIP-Binding5 | Forward | AAAAGGGAGGAGGGAGGC |
|  | Reverse | AATCAGCCACCCTTAGACGG |
| ChIP-Binding6 | Forward | CCAGCCACCACTTTCTCAAA |
|  | Reverse | GGGCGGTTTCAGAAAGACAA |
| ChIP-Binding7 | Forward | ACACTCCTCCCTCCGTCTC |
|  | Reverse | TGCCTTAATGAGATGCCGGA |
| ChIP-ctrl | Forward | TGCTGTGAAGATGGTTCAGT |
|  | Reverse | GCCATACGCTGTTGTCCAAA |
| ChIP-site1 | Forward | CTGGCCAACACAGTGAAAC |
|  | Reverse | AGTTTCACTCTGTCGCCCT |
| ChIP-site2 | Forward | GAGAGAGCGGGTATGAGGAC |
|  | Reverse | CCTGGAGCTGAGCAAGACA |

**Supplementary Table S2** Antibodies and reagents used in this study

|  | | | | |  |  |  |
| --- | --- | --- | --- | --- | --- | --- | --- |
| Antibodies and reagents | Vendors | Cat# | Working concentration (application) | |  |  |  |
| **Antibodies** |  |  |  | |  |  |  |
| GAPDH | ABclonal | AC001 | 1:5000 (WB) | |  |  |  |
| HIF2a | CST | #59973S | 1:1000 (WB),1:100(ChIP) | |  |  |  |
| TCF7L2 | CST | #2569 | 1:1000 (WB),1:50(IHC) | |  |  |  |
| ZEB1 | CST | #3396 | 1:1000 (WB) | |  |  |  |
| E-cadherin | CST | #14472 | 1:1000 (WB), 1:50(IF) | |  |  |  |
| N-cadherin | CST | #13116 | 1:1000 (WB) | |  |  |  |
| Vimentin | CST | #5741 | 1:1000 (WB), 1:100(IF) | |  |  |  |
| Snail | CST | #3879 | 1:1000 (WB) | |  |  |  |
| FASN | ABclonal | A0461 | 1:1000 (WB) | |  |  |  |
| ACC1 | ABclonal | A15606 | 1:1000 (WB) | |  |  |  |
| SCD | ABclonal | A16429 | 1:1000 (WB) | |  |  |  |
| H3K14ace | ABclonal | A7254 | 1:1000 (WB) | |  |  |  |
| H3K18ace | ABclonal | A20735 | 1:1000 (WB) | |  |  |  |
| H3K27ace | ABclonal | A7253 | 1:1000 (WB) | |  |  |  |
| Histone H3 | ABclonal | A2348 | 1:1000 (WB) | |  |  |  |
| METTL3 | ABclonal | A8370 | 1:1000 (WB) | |  |  |  |
| YTHDC1 | Abcam | ab122340 | 1:1000 (WB/IHC) | |  |  |  |
| N6-Methyladenosine | Beyotime | AF7407 | 1:100 (MeRIP) | |  |  |  |
| Anti-Mouse | ABclonal | AS003 | 1:2000 (WB) | |  |  |  |
| Anti-Rabbit | ABclonal | AS014 | 1:2000 (WB) | |  |  |  |
| **Reagents** |  |  |  | |  |  |  |
| Oleic acid | | | | | MCE | HY-N1446 | 20 µM |
| Wnt3a | MCE | HY-P70453A | | 20ng/mL |  |  |  |
| lithium acetoacetate | Sigma | A8509 | | 50 µM |  |  |  |
| Etomoxir | MCE | HY-50202 | | 50 µM |  |  |  |
| actinomycin D | MCE | HY-17559 | | 5 μg/mL |  |  |  |
|  |  |  | |  |  |  |  |

**Supplementary Table S3** siRNA and shRNA target sequences

| Gene |  | Sequences (5'- -3') |
| --- | --- | --- |
| siMETTL3 |  | CTGCAAGTATGTTCACTATGA |
| siYTHDC1 |  | GCAAGGAGUGUUAUCUUAATT |
| shHIF2a |  | CAGTACCCAGACGGATTTCAA |
| shTCF7L2 |  | CCCACATAAAGAAACCTCTTA |

**Supplementary Table S4** The results of SRAMP prediction based on TCF7L2 cDNA sequence

|  | Position | Sequence context (5'- -3') | Score |
| --- | --- | --- | --- |
|  | 561 | ATGACCTAGGCGCCAACGACGAACTGATTTCCTTCAAAGACGAGG | 0.596 |
|  | 1523 | CTCCATAGTTCAAAGCATCAGGACTCCAAAAAGGAAGAAGAAAAG | 0.643 |
|  | 1879 | ACTGTTCGGGCTTGACCGACAGACTTTATGGTGCAAACCGTGCAG | 0.67 |
|  | 2643 | TCGGATAGCTTAGTTTTAAAAGACTGATTAAAAAACAAAAAGAAA | 0.325 |
|  | 3394 | ACCCTGCGCCCTTAGGACCCGGACTGACCGTGTACAAAACTTTAC | 0.6 |

a) SRAMP (A sequence-based m6A modification site predictor, http://www.cuilab.cn/sramp);

b) The A in red color means the potential m6A modification site;

c) The score indicates how reliable the prediction is.
