# Supplementary material for: HIF2α Promotes Cancer Metastasis through TCF7L2-Dependent Fatty Acid Synthesis in ccRCC
Source: Research (Wash D C). 2024 Feb 22;7:0322. doi: 10.34133/research.0322 (PMC10882601; doi:10.34133/research.0322)
Supplement: Supplementary 2 — Files S1 to S3 [file research.0322.f2.zip › Supplementary file 2.docx]

**Supplementary file 2 Original blots of the western blotting**
