# Supplementary material for: HIF2α Promotes Cancer Metastasis through TCF7L2-Dependent Fatty Acid Synthesis in ccRCC
Source: Research (Wash D C). 2024 Feb 22;7:0322. doi: 10.34133/research.0322 (PMC10882601; doi:10.34133/research.0322)
Supplement: Supplementary 2 — Files S1 to S3 [file research.0322.f2.zip › Supplementary file 3.docx]

**Supplementary file 3**

**Supplementary figure**

**
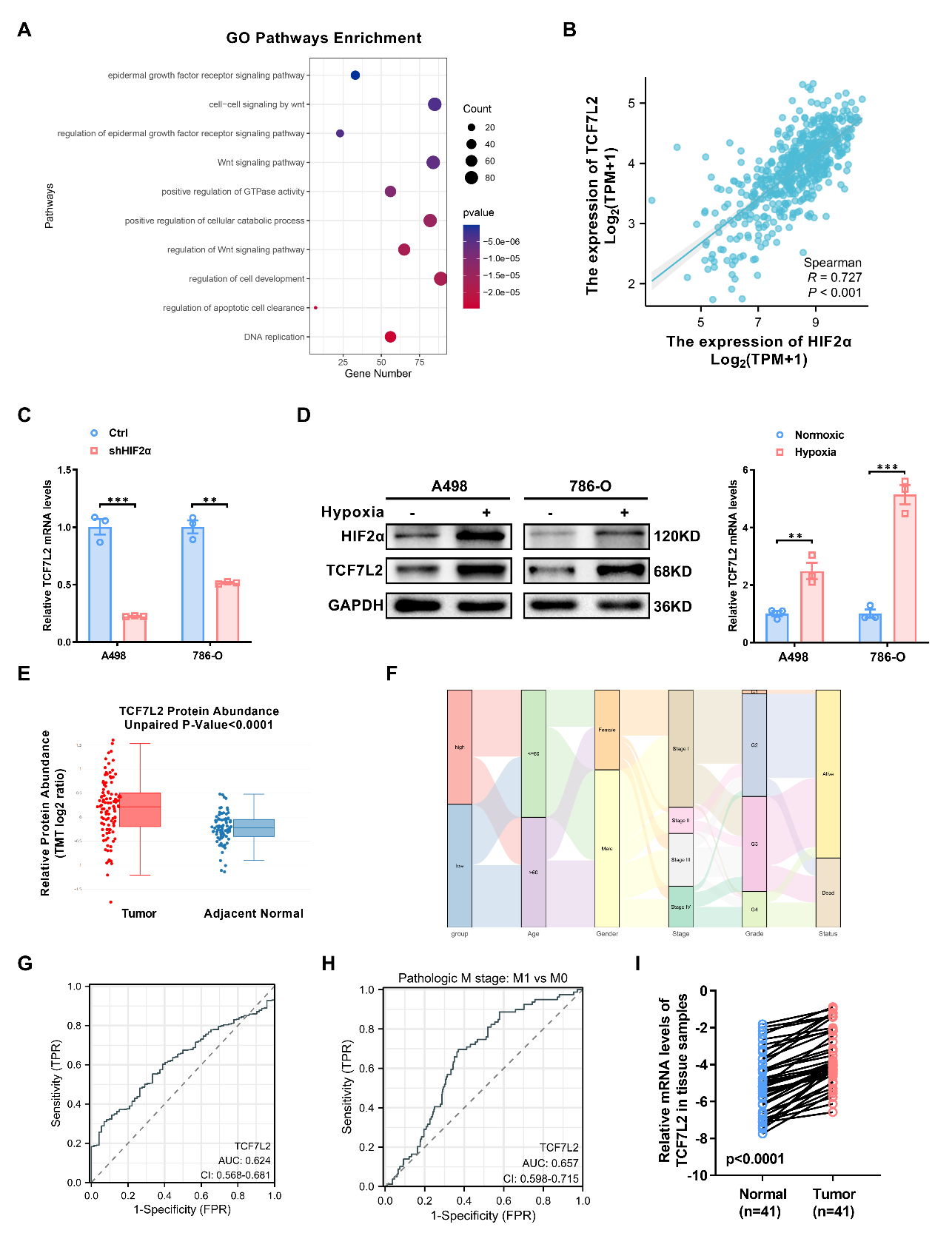
**

**Figure S1. HIF2α is associated with Wnt signaling in ccRCC.**

(A) A bubble plot depicting the gene ontology (GO) analysis of the intersection of differentially expressed genes from whole transcriptome sequencing data in HIF2α knockdown A498 and 786-O cells.

(B) Linear correlation curve between HIF2α and TCF7L2 based on TCGA-KIRC database data (statistical analysis based on Pearson correlation coefficient).

(C) qPCR analysis of TCF7L2 mRNA levels in HIF2α knockdown ccRCC cells (n=3). Statistical analysis was performed using independent sample t-test.

(D) Western blot and qPCR analysis of HIF2α and TCF7L2 proteins in ccRCC cells under hypoxic conditions (n=3).

(E) Protein expression of TCF7L2 in renal cancer and adjacent normal tissues as retrieved from the Proteomic Data Commons database.

(F) A Sankey diagram illustrating the correlation between TCF7L2 expression levels and clinical pathological features in ccRCC patients.

(G) ROC curve analysis of TCF7L2 in the TCGA-KIRC database.

(H) ROC curve analysis of TCF7L2 in the TCGA-KIRC database stratified by M0 and M1 groups.

(I) The levels of TCF7L2 mRNA in 41 ccRCC tissues and adjacent non-malignant tissues. Paired t-test, P < 0.0001.


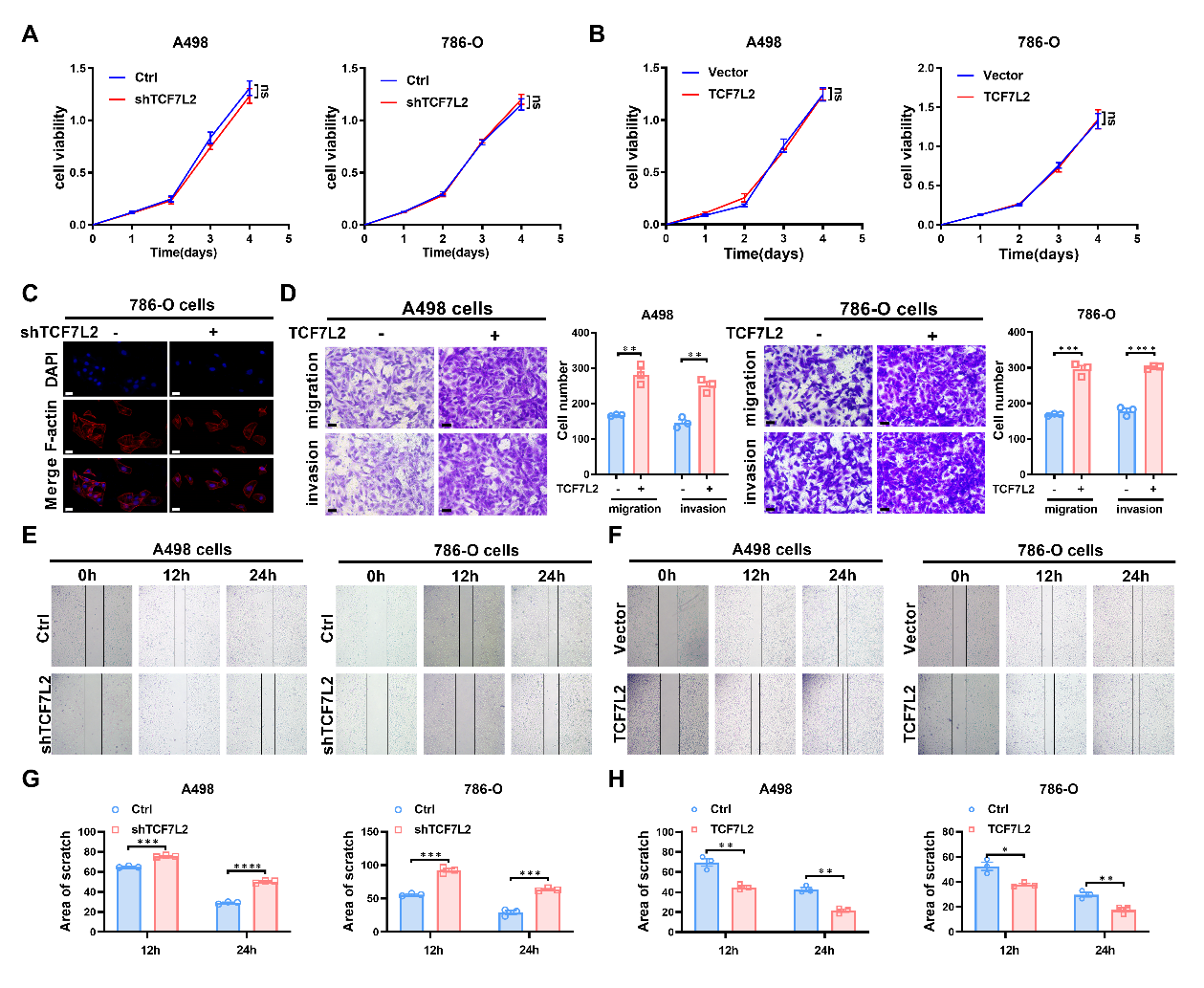


**Figure S2. TCF7L2 promotes metastasis in ccRCC.**

(A) Cell growth curve of TCF7L2 knockdown ccRCC cells measured by CCK-8 assay (independent sample t-test for statistical analysis).

(B) Cell growth curve of TCF7L2 overexpression ccRCC cells measured by CCK-8 assay (independent sample t-test for statistical analysis).

(C) Cell nuclei and cytoskeleton were stained using DAPI and F-actin, respectively ((n = 3, scale bar: 50 μm).

(D) Transwell assay results showing the migration and invasion of TCF7L2 overexpression ccRCC cells (n = 3, scale bar: 50 μm). Statistical analysis was performed using independent sample t-test.

(E) Scratch healing assay in TCF7L2 knockdown ccRCC cells (n=3).

(F) Scratch healing assay in TCF7L2 overexpression ccRCC cells (n=3).

(G) Statistical plot of scratch healing in TCF7L2 knockdown ccRCC cells (n=3). Statistical analysis was performed using independent sample t-test.

(H) Statistical plot of scratch healing in TCF7L2 overexpression ccRCC cells (n=3). Statistical analysis was performed using independent sample t-test.


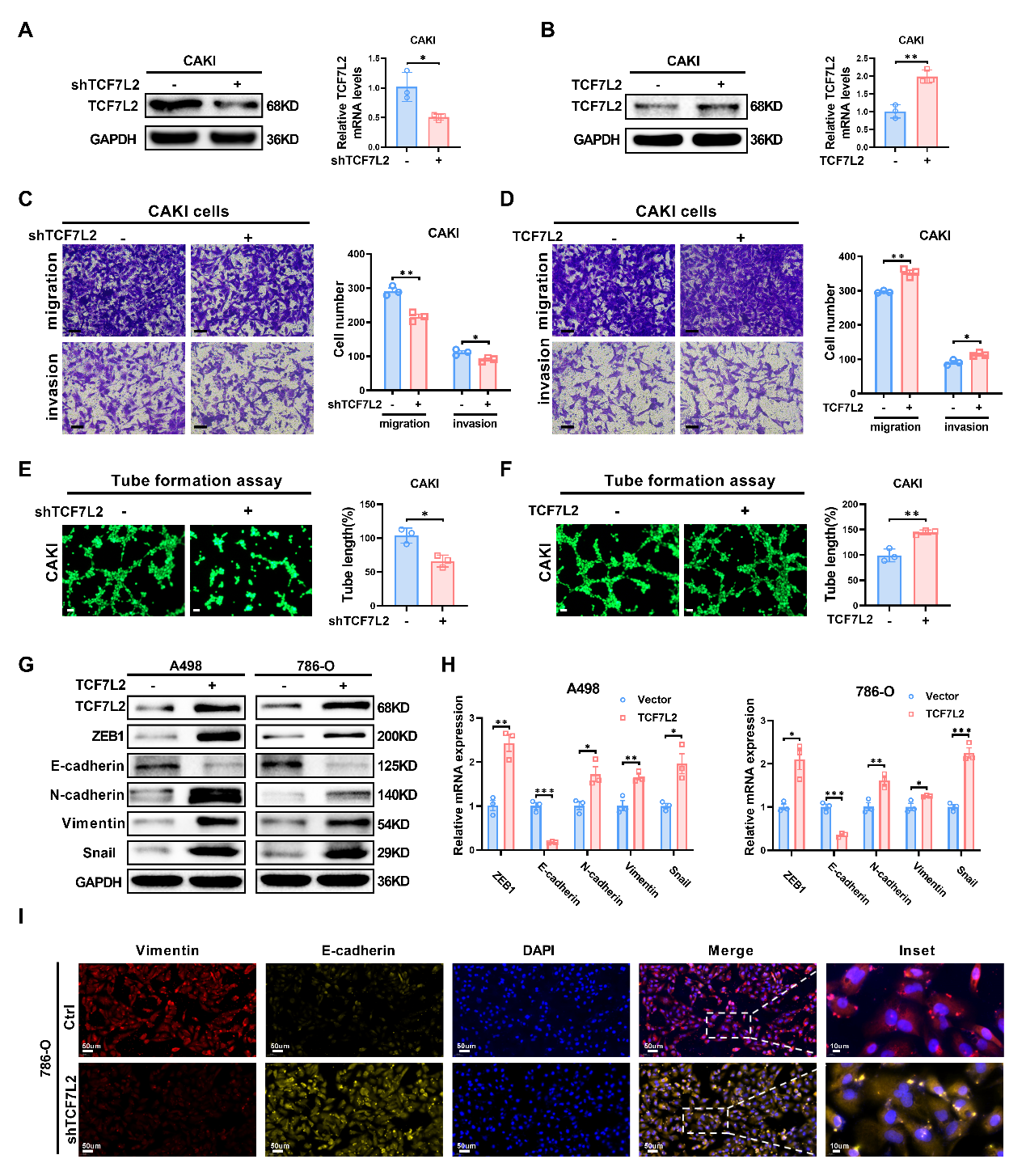


**Figure S3. TCF7L2 Activates the EMT Pathway in ccRCC.**

(A) The knockdown of TCF7L2 was validated at both the protein and mRNA levels using Western blotting and qPCR, respectively.

(B) The TCF7L2 overexpression was validated at both the protein and mRNA levels employing Western blotting and qPCR, respectively.

(C) Transwell assay results indicating the migration and invasion of TCF7L2 knockdown CAKI cells (n = 3, scale bar: 50 μm). Statistical analysis was conducted employing independent sample t-test.

(D) Transwell assay results showing the migration and invasion of TCF7L2 overexpression CAKI cells (n = 3, scale bar: 50 μm). Statistical analysis was performed using independent sample t-test.

(E) Effect of TCF7L2 knockdown in CAKI cells on tube formation assay in HUVECs (n=3). Statistical analysis was performed employing independent sample t-test.

(F) Effect of TCF7L2 overexpression in CAKI cells on tube formation assay in HUVECs (n=3). Statistical analysis was performed employing independent sample t-test.

(G) Western blot analysis of EMT markers in TCF7L2 overexpression ccRCC cells (n=3).

(H) qPCR analysis of EMT markers in TCF7L2 overexpression ccRCC cells (n=3). Statistical analysis was performed using independent sample t-test.

(I) Immunofluorescence staining of vimentin, E-cadherin, and DAPI in TCF7L2 overexpression ccRCC cells (n=3).

**
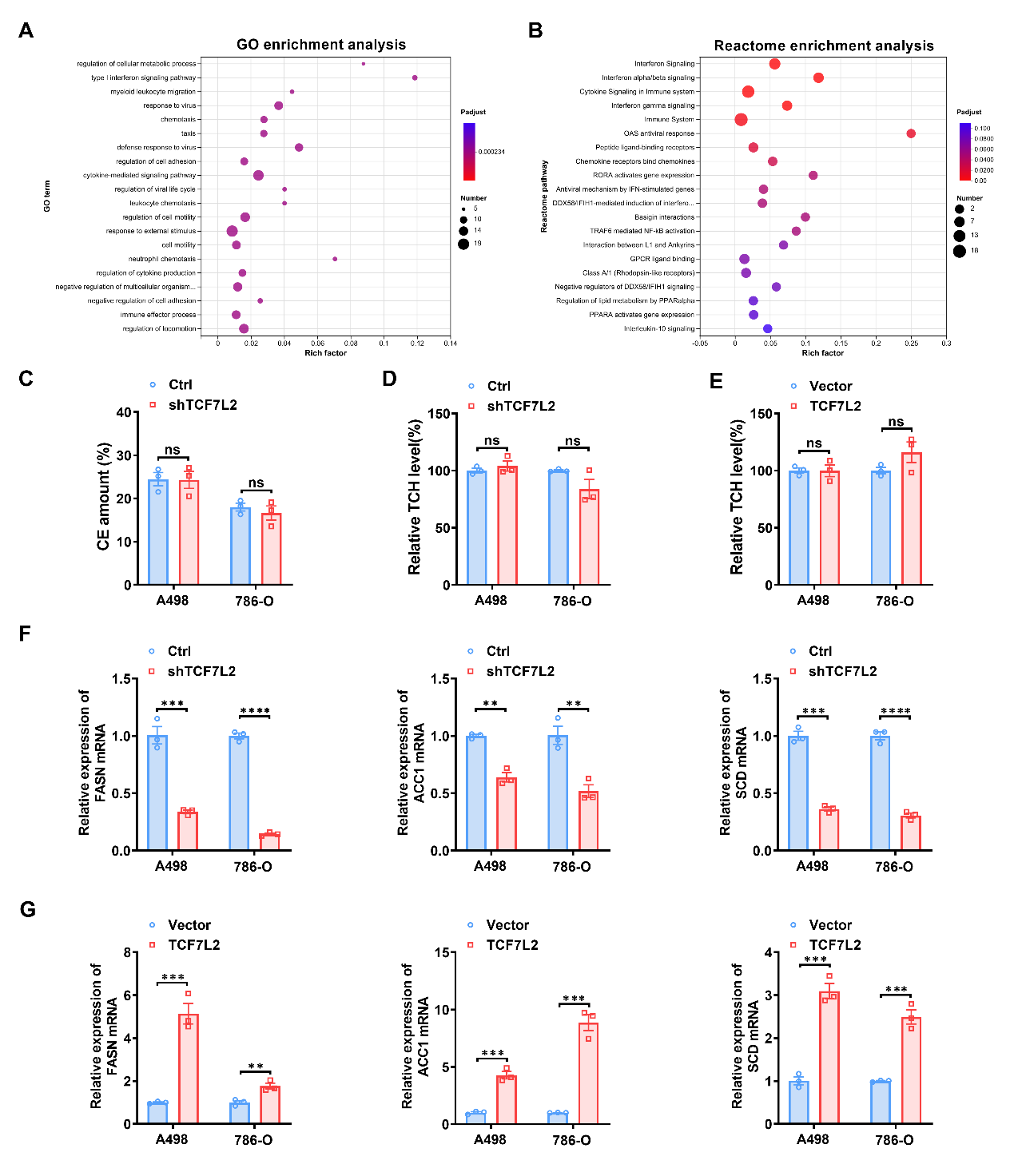
**

**Figure S4.** **TCF7L2 promotes fatty acid synthesis and oxidation in ccRCC.**

The results represent the mean ± SEM of three independent experiments with at least three replicates per experiment. ****P < 0.0001, ***P < 0.001, **P < 0.01, and *P < 0.05.

(A) GO enrichment analysis of differentially expressed genes identified by transcriptome sequencing.

(B) Reatome enrichment analysis of differentially expressed genes identified by transcriptome sequencing.

(C) Abundance of CEs in TCF7L2 knockdown ccRCC cells (n=3). Statistical analysis was performed using independent sample t-test.

(D-E) Relative levels of TCH in TCF7L2 knockdown or overexpressing ccRCC cell lines. Statistical analysis was performed using independent sample t-test.

(F-G) Evaluation of mRNA levels of lipid synthesis genes (FASN, ACC1, SCD) in TCF7L2 knockdown or overexpressing cell lines by qPCR (n = 3). Statistical analysis was performed using independent sample t-test.


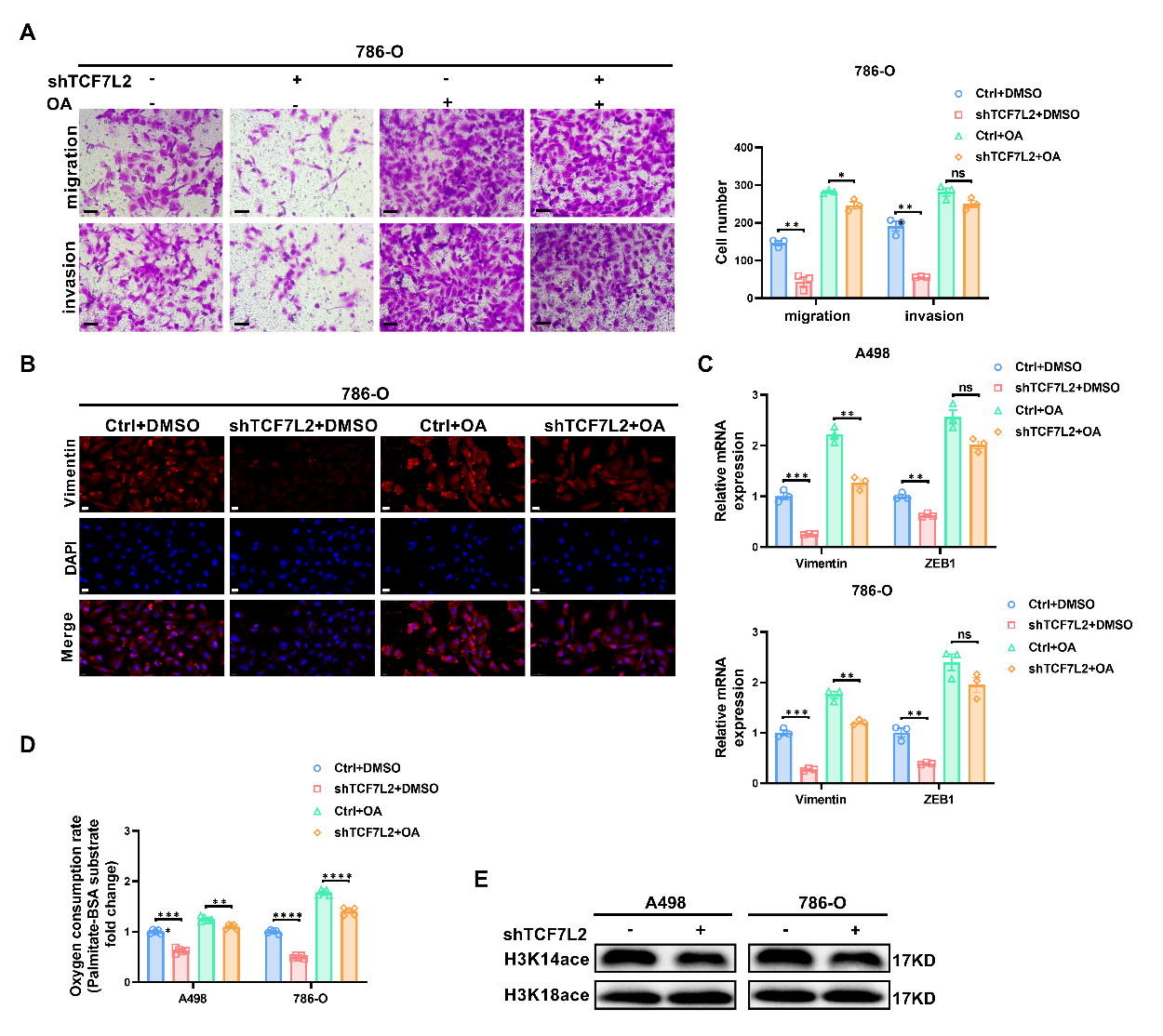


**Figure S5. EMT is controlled by TCF7L2-driven fatty acid metabolism.**

The results represent the mean ± SEM of three independent experiments with at least three replicates per experiment. ****P < 0.0001, ***P < 0.001, **P < 0.01, and *P < 0.05.

(A) Transwell assay results showing the migration and invasion of Ctrl+DMSO, shTCF7L2+DMSO, Ctrl+OA, shTCF7L2+OA 786-O cells (n = 3, scale bar: 50 μm). Statistical analysis was performed using independent sample t-test.

(B) Immunofluorescence staining of Vimentin and DAPI of Ctrl+DMSO, shTCF7L2+DMSO, Ctrl+OA, shTCF7L2+OA 786-O cells (n=3, scale bar: 20μm).

(C) qPCR analysis of EMT markers of Ctrl+DMSO, shTCF7L2+DMSO, Ctrl+OA, shTCF7L2+OA ccRCC cells (n=3). Statistical analysis was performed using independent sample t-test.

(D) Measurement of OCR levels in the Ctrl+DMSO, shTCF7L2+DMSO, Ctrl+OA, shTCF7L2+OA groups of ccRCC cells. Statistical analysis was performed using independent sample t-test.

(E) Protein expression of H3K14 and H3K18 acetylation in TCF7L2 knockdown and negative control ccRCC cells.

**
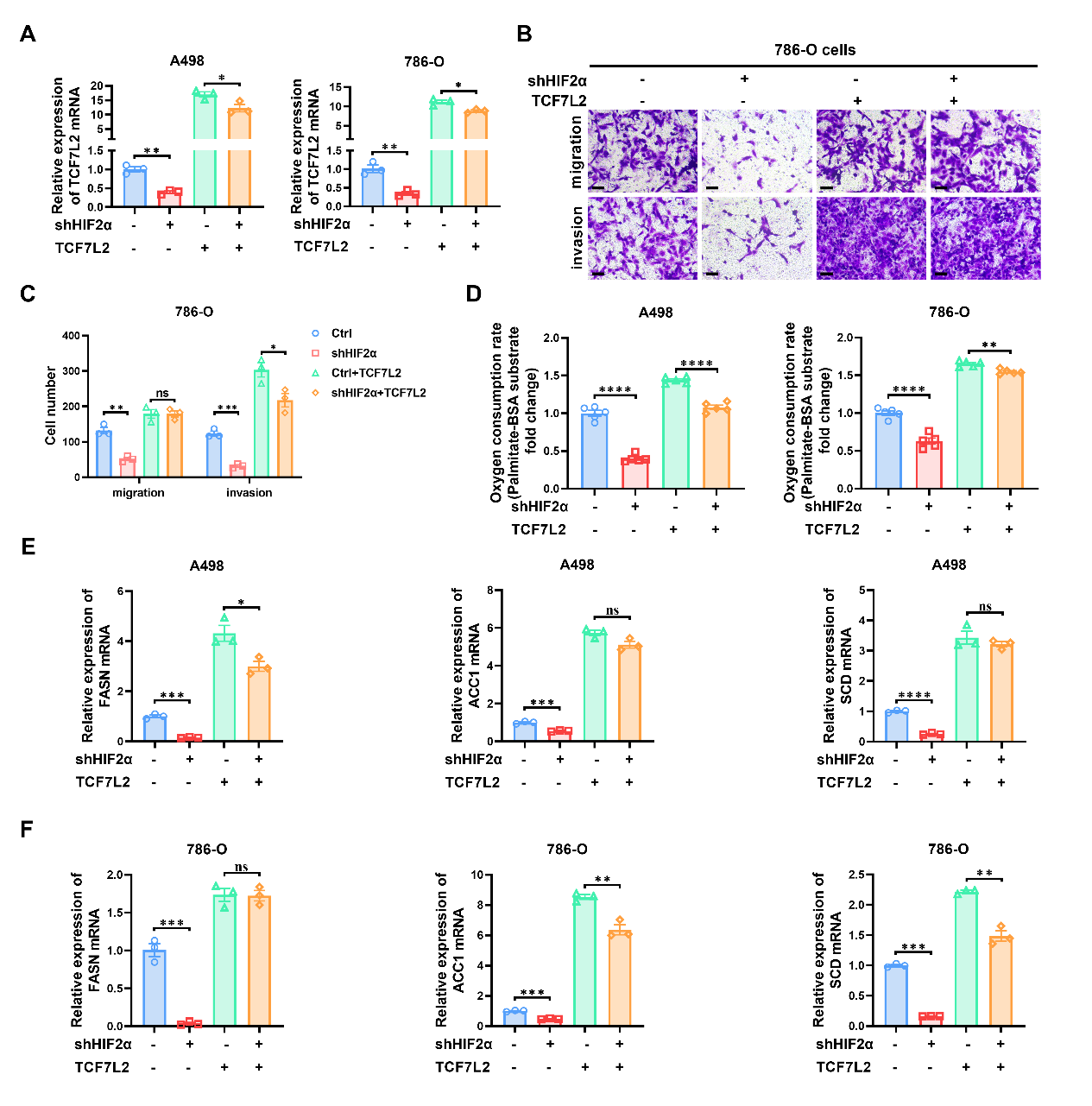
**

**Figure S6. TCF7L2 mediates the effects of HIF2α on ccRCC metastasis and lipid metabolism.**

(A) qPCR analysis revealed the mRNA levels of HIF2α and TCF7L2 in the functional recovery cell lines (n = 3). Statistical analysis was performed using independent sample t-test.

(B) Transwell assay images of the functional recovery 786-O cell lines (n = 3, scale bar: 50 μm).

(C) Quantification of Transwell assay for the functional recovery 786-O cell lines (n = 3). Statistical analysis was performed using independent sample t-test.

(D) Measurement of OCR levels in the functional recovery cell lines (n = 3). Statistical analysis was performed using independent sample t-test.

(E-F) qPCR analysis of lipid synthesis-related genes FASN, ACC1, and SCD in the functional recovery ccRCC cell lines (n = 3). Statistical analysis was performed using independent sample t-test.

**
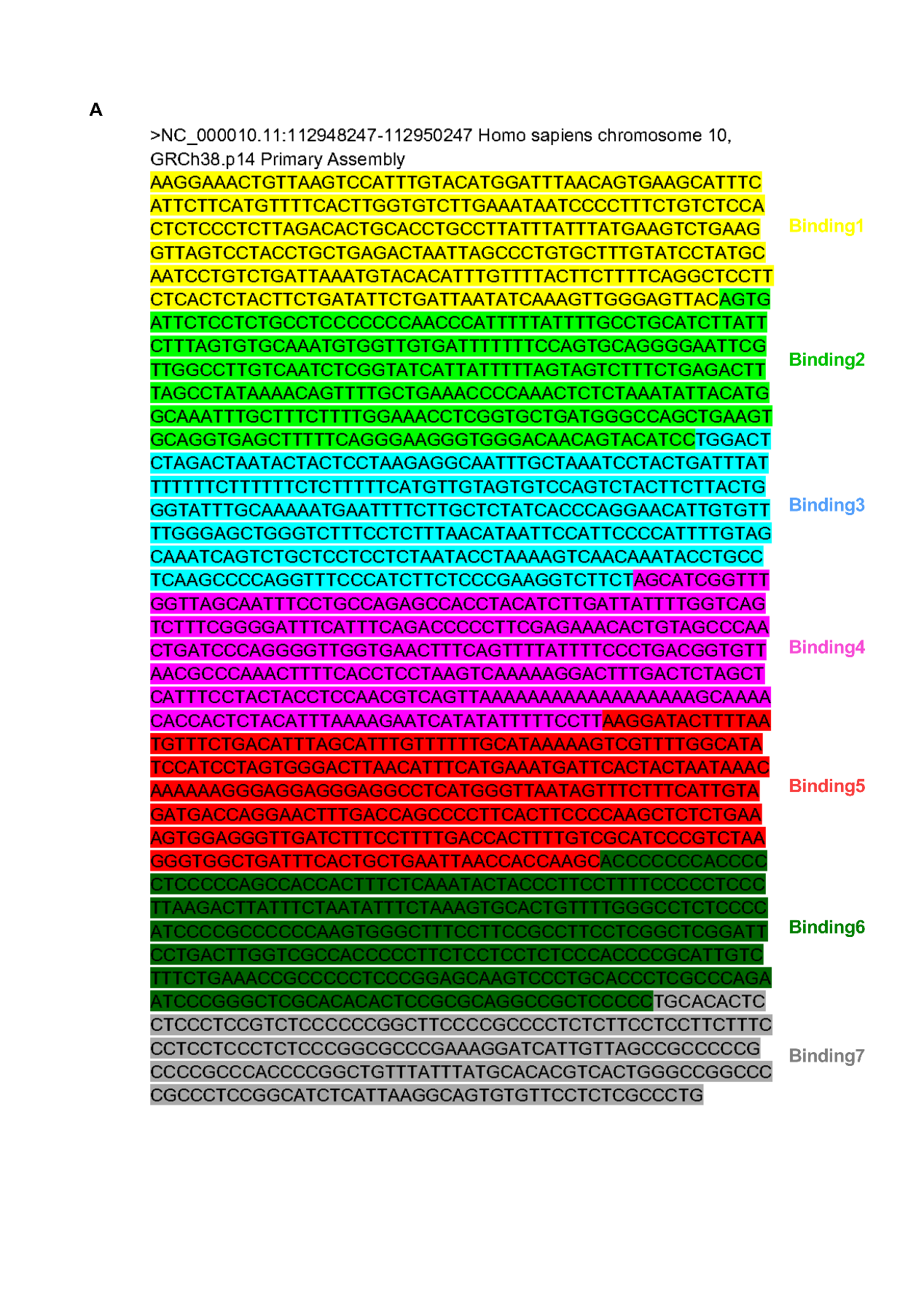
**

**Figure S7. HIF2α does not directly transcribe to activate TCF7L2 expression.**

(A) Seven binding sequences were delineated based on the division of the 2,000 bp upstream promoter region of the TCF7L2 transcription start site.

**
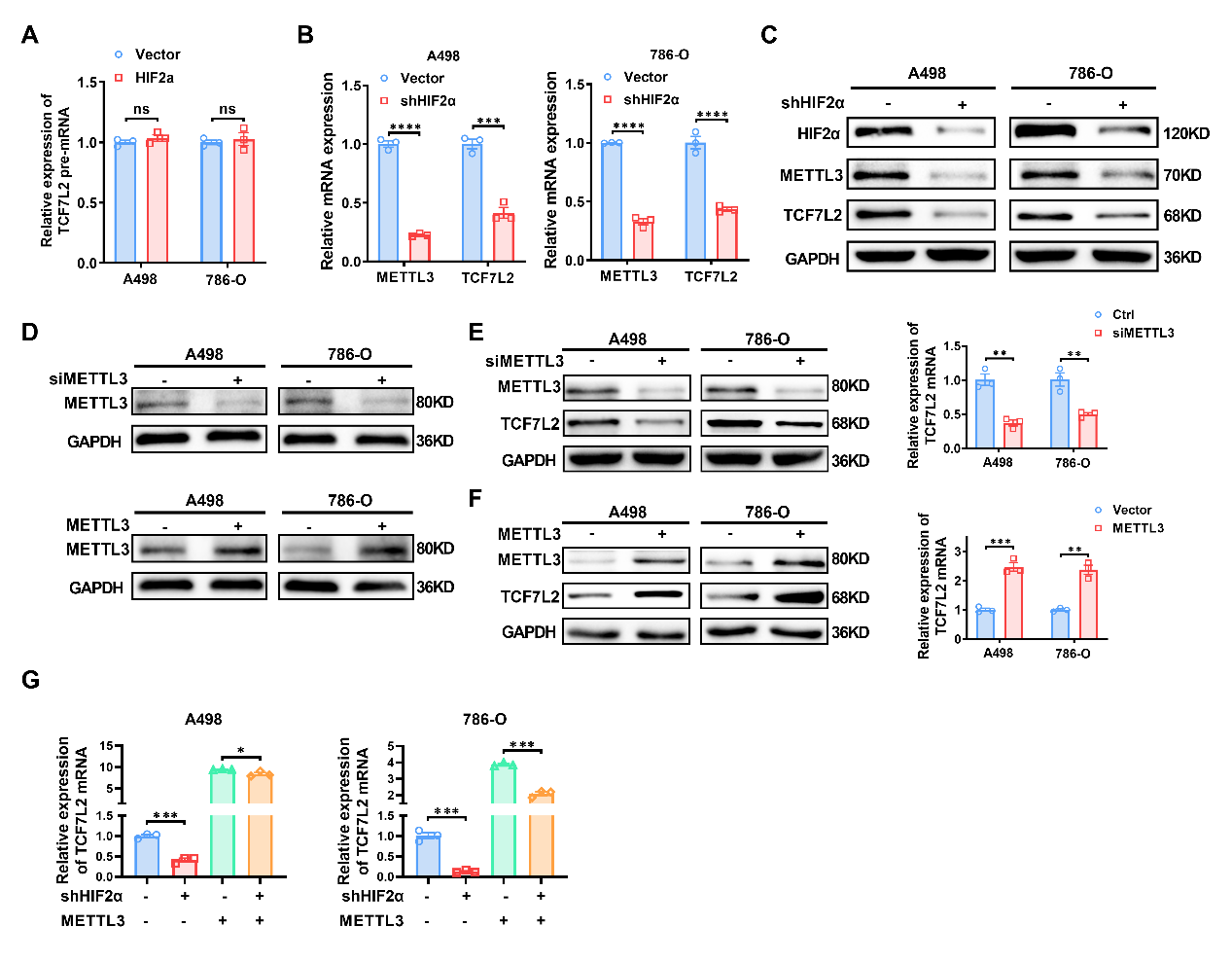
**

**Figure S8. HIF2α transcriptionally activates METTL3 to stabilize TCF7L2 mRNA.**

The results represent the mean ± SEM of three independent experiments with at least three replicates per experiment. ****P < 0.0001, ***P < 0.001, **P < 0.01, and *P < 0.05.

(A) Levels of TCF7L2 pre-mRNA in HIF2α knockdown and control ccRCC cells (n=3). Statistical analysis was conducted employing independent sample t-test.

(B) qPCR analysis revealed the mRNA levels of METTL3 and TCF7L2 in HIF2α knockdown ccRCC cells (n = 3). Statistical analysis was performed using independent sample t-test.

(C) Western blot analysis of METTL3 and TCF7L2 in HIF2α knockdown and overexpression ccRCC cells (n=3).

(D) Western blot analysis of METTL3 in METTL3 knockdown or overexpression ccRCC cells (n=3).

(E) Western blot and qPCR analysis of METTL3 and TCF7L2 in METTL3 knockdown ccRCC cells (n=3).

(F) Western blot and qPCR analysis of METTL3 and TCF7L2 in METTL3 overexpression ccRCC cells (n=3).

(G) qPCR analysis reveals the mRNA levels of TCF7L2 in the Ctrl, shHIF2α, Ctrl+METTL3, and shHIF2α+METTL3 groups (n = 3).


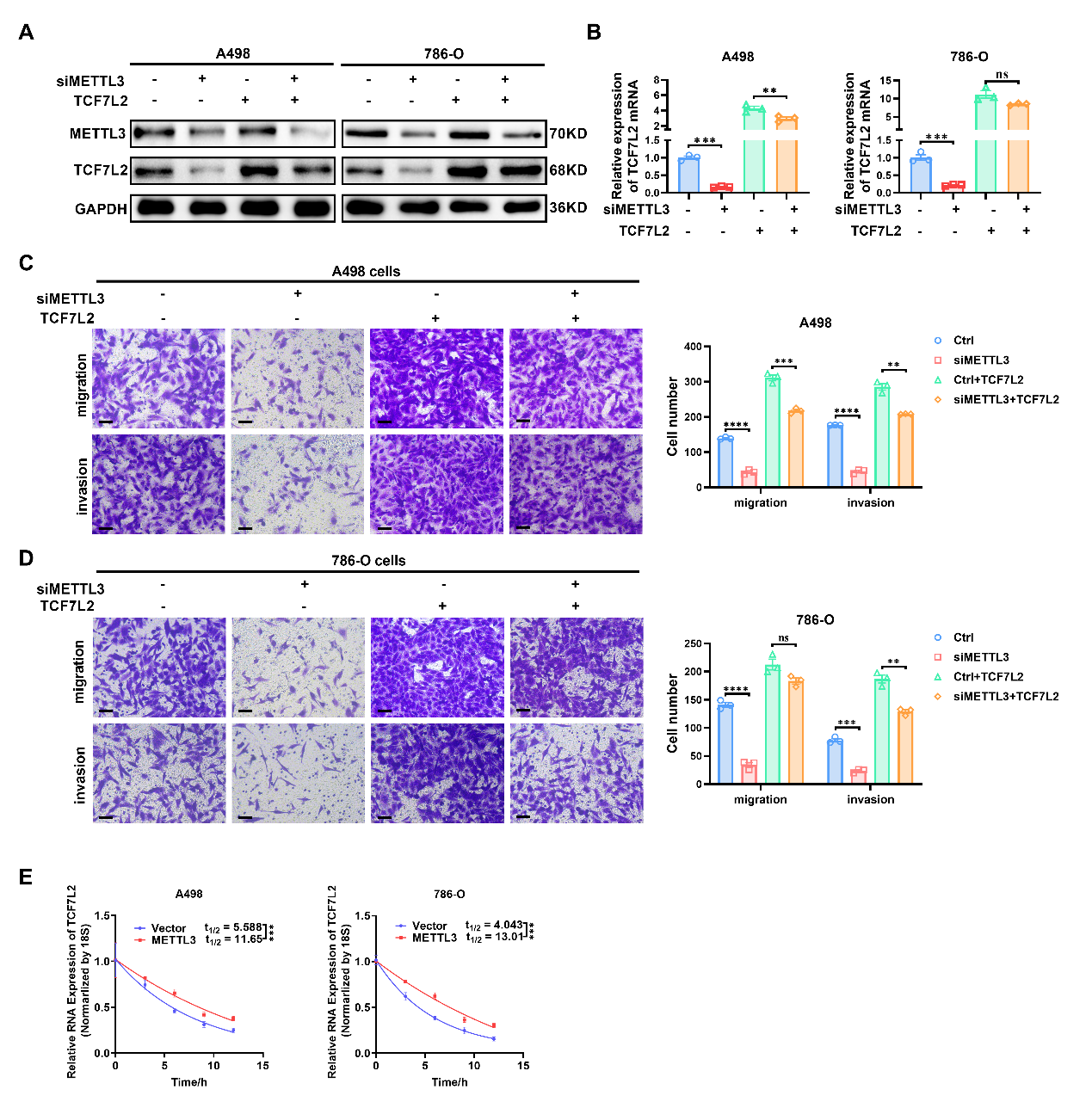


**Figure S9. METTL3 promotes ccRCC metastasis by enhancing the stability of TCF7L2 mRNA.**

(A) Western blot analysis reveals the protein expression levels of METTL3 and TCF7L2 in the Ctrl, siMETTL3, Ctrl+TCF7L2, and siMETTL3+TCF7L2 groups (n = 3).

(B) qPCR analysis demonstrates the mRNA expression levels of TCF7L2 in the Ctrl, siMETTL3, Ctrl+TCF7L2, and siMETTL3+TCF7L2 groups (n = 3).

(C-D) Transwell experiment images and quantitative analysis for the Ctrl, siMETTL3, Ctrl+TCF7L2, and siMETTL3+TCF7L2 groups.

(E) Decay rates of TCF7L2 mRNA after METTL3 overexpression in A498 and 786-O cells treated with Actinomycin D (n=3). Statistical analysis was conducted employing independent sample t-test.


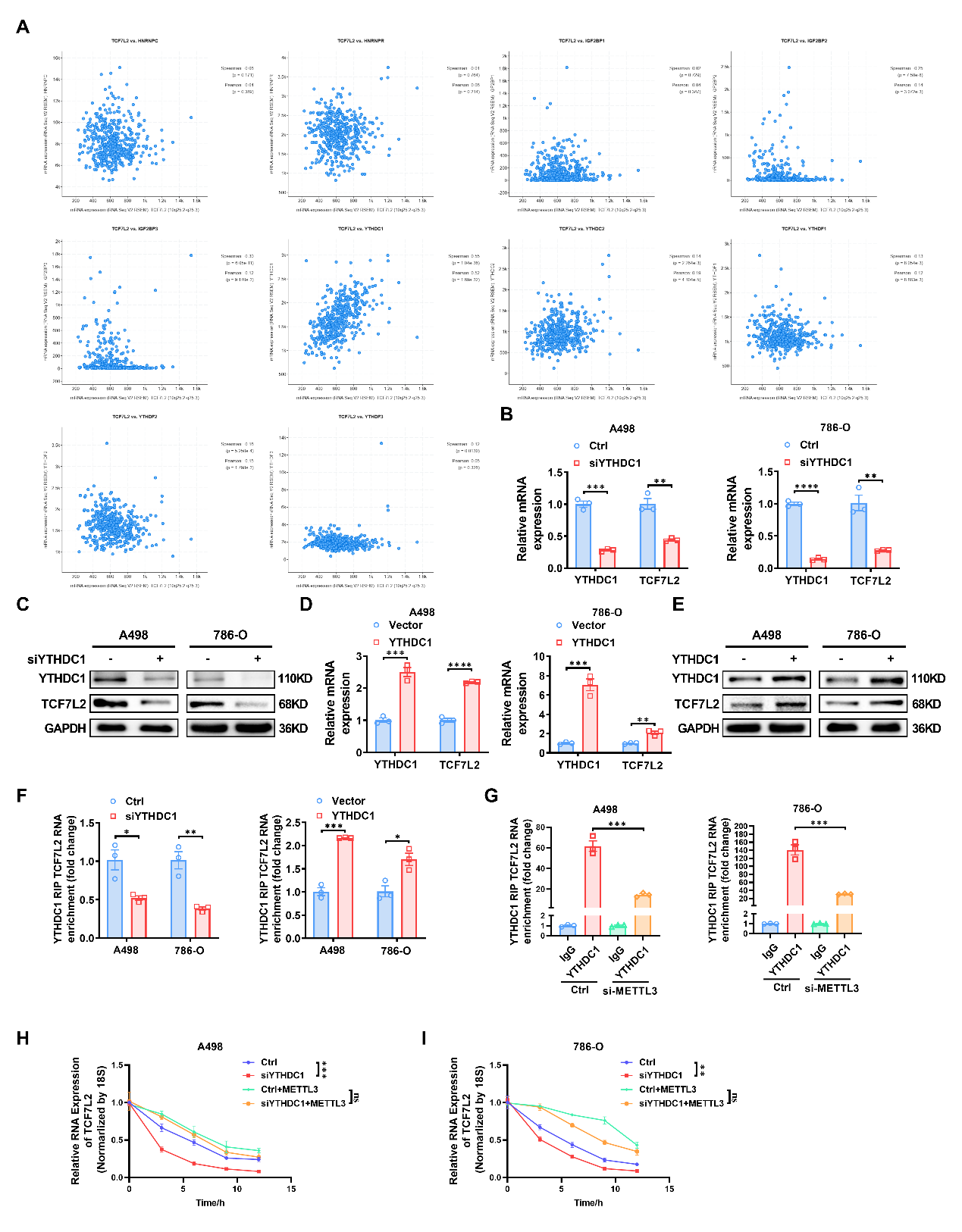


**Figure S10. The stability of TCF7L2 mRNA is regulated through the METTL3-YTHDC1 axis.**

(A) Linear correlation curves (based on statistical analysis using Spearman and Pearson correlation coefficients) depicting the relationships between TCF7L2 and HNRNPC, HNRNPR, IGF2BP1, IGF2BP2, IGF2BP3, YTHDC1, YTHDC2, YTHDF1, YTHDF2, and YTHDF3, based on data from the TCGA-KIRC database.

(B) qPCR analysis revealed the mRNA levels of YTHDC1 and TCF7L2 in YTHDC1 knockdown ccRCC cells (n = 3). Statistical analysis was performed using independent sample t-test.

(C) Western blot analysis of YTHDC1 and TCF7L2 protein expression levels in ccRCC cells with YTHDC1 knockdown (n = 3).

(D) qPCR analysis revealed the mRNA levels of YTHDC1 and TCF7L2 in YTHDC1 overexpression ccRCC cells (n = 3). Statistical analysis was performed using independent sample t-test.

(E) Western blot analysis of YTHDC1 and TCF7L2 protein expression levels in ccRCC cells with YTHDC1 overexpression (n = 3).

(F) RIP-qPCR was conducted to relative quantify the binding levels of TCF7L2 mRNA following YTHDC1 knockdown or overexpression. Statistical analysis was performed using independent sample t-test.

(G) RIP-qPCR was utilized to assess the relative quantity of TCF7L2 mRNA binding to YTHDC1 in siMETTL3-treated cells compared to control cells. Statistical analysis was performed using independent sample t-test.

(H-I) Decay rates of TCF7L2 mRNA in A498 or 786-O cells treated with actinomycin D in Ctrl, siYTHDC1, Ctrl+METTL3, and siYTHDC1+METTL3 groups (n=3). Statistical analysis was performed using independent sample t-test.


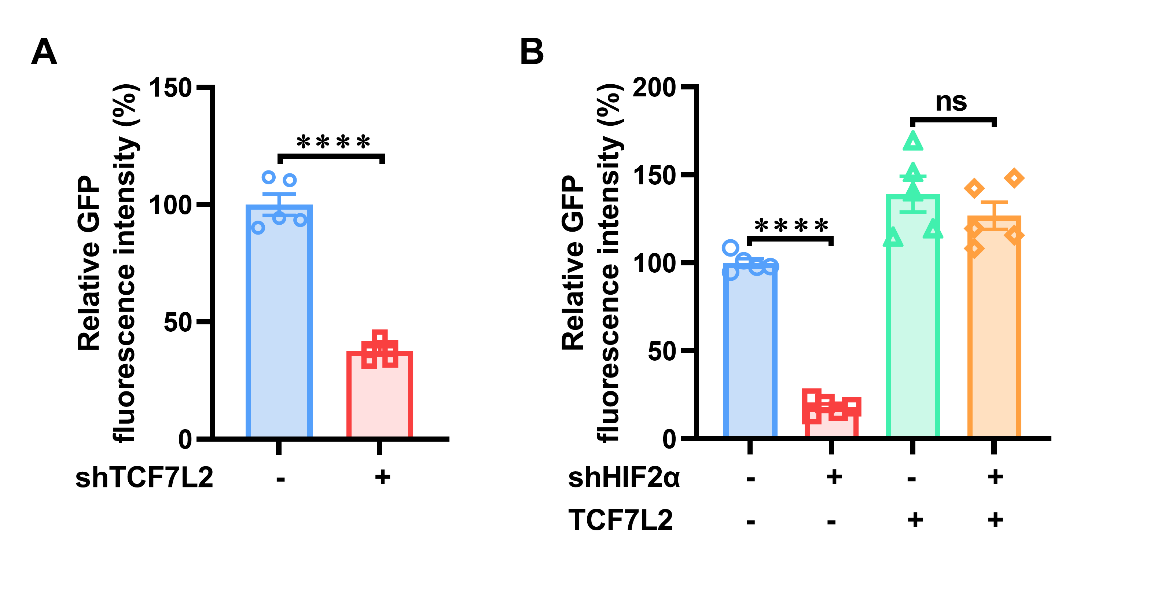


**Figure S7. TCF7L2, as a target gene of HIF2α, promotes ccRCC metastasis in vivo.** The results represent the mean ± SEM of three independent experiments with at least three replicates per experiment. ****P < 0.0001, ***P < 0.001, **P < 0.01, and *P < 0.05.

(A) Quantification of GFP fluorescence intensity in live imaging of metastatic tumor model mice constructed with TCF7L2 knockdown and negative control A498 cells (n = 5). Statistical analysis was performed using independent sample t-test.

(B) Quantification of GFP fluorescence intensity in live imaging of metastatic tumor model mice constructed with functional recovery cells (n = 5). Statistical analysis was performed using independent sample t-test.
